# A Remote Intervention Based on mHealth and Community Health Workers for Antiretroviral Therapy Adherence in People With HIV: Pilot Randomized Controlled Trial

**DOI:** 10.2196/67997

**Published:** 2025-04-02

**Authors:** Shivesh Shourya, Jianfang Liu, Sophia McInerney, Trinity Casimir, James Kenniff, Trace Kershaw, David Batey, Rebecca Schnall

**Affiliations:** 1 Department of Social and Behavioral Sciences Yale School of Public Health New Haven, CT United States; 2 Columbia University School of Nursing New York, NY United States; 3 Tulane University School of Social Work New Orleans, LA United States

**Keywords:** HIV, antiretroviral therapy adherence, ART, ART adherence, mobile health technology, mHealth technology, community health workers, CHWs, smart pill bottle, feasibility evaluation, mobile health technology acceptance model

## Abstract

**Background:**

Despite the availability of antiretroviral therapy (ART), only 66% of people with HIV in the United States achieve viral suppression, largely due to suboptimal ART adherence. Barriers such as limited access to care and forgetfulness impact adherence rates, which must be maintained at ≥95% to prevent viral load rebound. Combination interventions leveraging community health worker (CHW) support and mobile health (mHealth) technologies have the potential to overcome previously identified barriers and provide cost-effective support for improving adherence and viral suppression outcomes in people with HIV.

**Objective:**

This pilot study aimed to evaluate the feasibility, acceptability, and preliminary efficacy of remote delivery of the Community Health Worker and mHealth to Improve Viral Suppression (CHAMPS) intervention, combining the WiseApp, CHW support, and the CleverCap smart pill bottle. A secondary aim was to gather participants’ feedback on the usability of the app and pill bottle as well as to better understand their experiences with remote study procedures.

**Methods:**

This mixed methods pilot study involved 40 participants with HIV, who were randomly assigned to a control group (n=20, 50%) or the CHAMPS intervention (n=20, 50%) over 3 months. The intervention group participated in up to 12 sessions with CHWs and used the WiseApp, paired with a CleverCap smart pill bottle, to support ART adherence. Remote baseline and follow-up visits were conducted via Zoom and included surveys measuring adherence, self-efficacy, and usability (measured by Health Information Technology Usability Evaluation Scale [Health-ITUES] and Poststudy System Usability Questionnaire [PSSUQ]). Semistructured interviews explored participants’ experiences with the intervention. Thematic analysis was used to identify key facilitators and barriers based on the Mobile Health Technology Acceptance Model.

**Results:**

Remote delivery of the CHAMPS intervention was feasible, with high usability ratings for both the WiseApp and CleverCap (overall scores on Health-ITUES: mean 4.35, SD 0.58 and PSSUQ: mean 2.04, SD 1.03). In the intervention group, there were nonsignificant improvements in self-reported adherence scores (*P*=.29) and in self-efficacy scores (*P*=.07). The adjusted odds ratio for achieving undetectable viral load in the intervention group compared to the control group was 3.01 (95% CI –1.59 to 4.12), indicating a medium effect size in favor of the intervention. Overall study retention was 75% (30/40), with higher retention in the control group. Participants valued the flexibility of remote study procedures, particularly Zoom-based study visits and mailed blood sample kits. Qualitative feedback highlighted the intervention’s acceptability and ability to overcome logistical barriers.

**Conclusions:**

The remote CHAMPS pilot study demonstrated the feasibility and acceptability of combining mHealth tools with CHW support to promote medication adherence among people with HIV. While further optimization is needed to enhance its impact, this intervention shows potential for improving health outcomes in diverse underserved populations.

**Trial Registration:**

ClinicalTrials.gov NCT05938413; https://clinicaltrials.gov/study/NCT05938413

## Introduction

### Background

With the advent of antiretroviral therapy (ART) for HIV, people with HIV can now achieve near-normal life expectancies [1-4]. At the end of 2021, >50% of people with HIV in the United States were aged ≥50 years, primarily due to the effectiveness of ART [5]. However, the rates of viral suppression among the 1.2 million people with HIV in the United States continue to remain low, with current estimates of approximately 66% among people with HIV, despite the wider availability of ART [5]. The suboptimal rates of viral suppression may be attributed to poor ART adherence [6] and occur during a period of increasing health care fragmentation, leading to higher costs of care and lower health outcomes for many patients with chronic illnesses, such as people with HIV [7].

The Ending the HIV Epidemic plan identifies viral suppression as a cornerstone to preventing and treating HIV infections [8-10]. HIV viral suppression is dependent on adherence to ART, and studies demonstrate that high rates of ART adherence (approximately 95%) are attributed to higher rates of viral suppression (approximately 78%) [11]. However, even moderate decreases in adherence to ART (from 95% to 80%) can lead to drastic reductions in viral suppression rates (from 78% to 20%), underscoring that ART adherence is maintained at ≥95% [11]. Poor adherence to ART can lead to poor outcomes for the patient, the emergence of drug-resistant HIV strains [12], and increased rates of HIV transmission [13]. Suboptimal levels of ART adherence, along with low levels of engagement in the HIV care continuum, have also been attributed to the progression of HIV disease and premature deaths among people with HIV [14]. Therefore, there is an urgent need to develop and evaluate interventions that enhance adherence to ART.

Developing interventions to increase ART adherence must address prevalent barriers to optimal ART adherence by championing a comprehensive approach to understanding and addressing the unmet needs of people with HIV. Previous studies have identified numerous barriers to sustained adherence to ART; engagement with medical care; and, consequently, viral suppression, including HIV stigma or fear of HIV status disclosure [15-19], geographic barriers or limited time resources for receiving or accessing HIV care [16,17,19], negative experiences within medical institutions or HIV care centers [15,16,19], and forgetfulness [18,19]. Similar studies have outlined strong networks of social support, HIV case managers, telemedicine appointments, and reminder tools as facilitators of ART adherence and medical care engagement [16-19]. Recently, there has been an increased focus on the utility of community health workers (CHWs), who can take on roles as outreach workers, patient navigators, health advisers, or peer leaders in helping patients access and manage HIV in primary care settings [20]. While the profile, skill level, and job scope of CHWs can vary widely based on context, they often serve as patient navigators and peer educators in the United States, focusing on HIV care retention, ART adherence, and viral suppression support, particularly in underresourced communities [21-23]. Several studies have found that integrating CHWs was associated with improved care retention, ART adherence, and viral suppression outcomes [20,24,25]; nonetheless, other studies have found no significant difference in viral suppression outcomes between people with HIV with assigned CHWs and those without CHW interventionists [26,27]. While some studies failed to show statistical significance in viral suppression outcomes between intervention and control groups, they emphasized the utility of a combined intervention, including CHW components and other interventions, to address barriers to ART adherence [26,27]. Mobile health (mHealth) interventions offer themselves as novel interventions in HIV care settings, given their ubiquity, ability to overcome geographic barriers to care, and low associated costs [28]. However, previous studies have provided mixed results, with some finding increased viral suppression and retention in care rates among mHealth intervention participants [29,30] and others finding no statistically significant difference in viral suppression and retention rates between intervention and control participants [31]. Previous study limitations include poor integration of mHealth technologies within HIV primary care settings, and researchers have advocated for a combination approach with patient navigation services to help prioritize human communications and achieve desired outcomes related to viral suppression [31]. Thus, interventions that combine the use of CHWs and mHealth technologies hold promise for increasing rates of ART adherence and viral suppression within the United States [24,32].

### Objectives

In this pilot study of the Community Health Worker and Mobile Health to Improve Viral Suppression (CHAMPS) remote intervention, conducted by our team at the Columbia University School of Nursing, we tested the feasibility of a remotely delivered CHW intervention through the mHealth app WiseApp. Participants were recruited from across the United States and provided with a smart pill bottle, CleverCap, which interfaced with the WiseApp for personalized medication reminders [33,34]. We also gathered feedback on the acceptability of remotely conducted study procedures.

## Methods

### Study Design

#### Overview

The pilot study was a 2-arm randomized controlled trial among 40 people with HIV who were followed over a 3-month period. Participants were randomly assigned to the CHAMPS intervention (20/40, 50%) or the standard of care control (20/40, 50%) arm. Recruitment for the study occurred over a 7-month period, beginning in July 2023 and closing at the end of January 2024. The study was initiated on July 26, 2023, with the enrollment of the first participant, and concluded in May 2024, with the final participant completing their last study visit on May 7, 2024.

#### Intervention Arm and Description of the Planned Intervention

The CHAMPS intervention was a 3-month intervention guided by the study team’s previous work on CHW and mHealth interventions. Specifically, the Birmingham Access to Care study (NCT03205982) adapted the Anti-Retroviral Treatment and Access to Services intervention, an evidence-based intervention designated by the Centers for Disease Control and Prevention, to support re-engagement in care for people with HIV who had dropped out of care [34,35]. This approach emphasized strength-based case management and motivational interviewing, fostering a close, supportive relationship between CHWs and participants, and was used to design the content structure of the CHW sessions. In addition, the WiseApp study leveraged end user feedback to develop a self-management app for people with HIV, incorporating features such as medication trackers, push notification reminders, and linkage to the CleverCap smart pill bottle [34,36].

Participants in the intervention arm were assigned a CHW at the end of their baseline visit. CHWs were study team members trained on the intervention, including the content of each session, motivational interviewing, strength-based case management, Anti-Retroviral Treatment and Access to Services [37], HIV and substance use, the WiseApp and associated mHealth technology, and field safety. CHWs administered at least 10 or up to 12 individual sessions to the participants throughout the intervention. Table 1 provides an outline and description of CHW session contents and structure. All sessions were conducted remotely via secure Zoom (Zoom Video Communications, Inc) calls, in compliance with the Health Insurance Portability and Accountability Act (HIPAA); phone calls; or the chat feature of the WiseApp, depending on the participant’s preference.

**Table 1:** Structure and content of the community health worker (CHW) sessions delivered to participants in the Community Health Worker and Mobile Health to Improve Viral Suppression (CHAMPS) intervention arm.

Session number and title	Session content	Window for session
(1) Building the relationship	Introduce the goals of the CHAMPS intervention to the participant (approximately 15 to 20 minutes).	At baseline visit or within 1 to 2 days of baseline visit
(2) Introduction to the WiseApp	Discuss how the WiseApp can be used to facilitate communication between the CHW and the participant. Review the medication tracking function and how this can be used by the CHW and the participant (approximately 30 to 45 minutes).	At baseline visit or within 1 to 2 days of baseline visit
(3) Emphasizing personal strengths	Help the participant self-identify personal strengths, abilities, and skills. Check in on any technical issues and study logistics with the participant (approximately 30 to 45 minutes).	Week 1
(4) Learning to make contact	Assist the participant in preparing a list of questions to ask their care provider. Check in on any technical issues and study logistics with the participant (approximately 30 to 45 minutes).	Week 2
(5) Check-in call 1	Check in with the participant on their upcoming appointments and any needed documents; address any potential barriers to care through offering local resources. Check in on any technical issues and study logistics with the participant (approximately 15 to 20 minutes).	Week 3
(6) Primary care provider appointment 1	Support the participant’s efforts during their primary care provider remote visit, on the basis of the participant’s comfort level (approximately 15 to 20 minutes).	On the basis of any upcoming appointments
(7) Debriefing the provider visit 1	Solicit the participant’s input on what went well during their primary care provider visit. Elicit from the participant what was learned from the care visit and what strengths they demonstrated. Check in on any technical issues and study logistics with the participant (approximately 15 to 20 minutes).	At the time of or within 1 to 2 days of session 6
(8) Reviewing progress	Plan for and create an action plan for the transition process as the participant nears the end of the study period and prepares to engage with standard-of-care case manager and related clinical professionals (eg, social worker). Check in with the participant on their upcoming appointments and any needed documents. Check in on any technical issues and study logistics with the participant (approximately 30 to 45 minutes).	Anytime during weeks 4 to 7
(9) Check in call 2	Check in with the participant on their upcoming appointments and any needed documents; address any potential barriers to care through offering local resources. Check in on any technical issues and study logistics with the participant (approximately 15 to 20 minutes).	Anytime during weeks 8 to 10
(10) Completing the work	Review the transition process action plan with the participant. Begin transition to standard-of-care case manager and related clinical professionals (eg, social worker) (approximately 30 to 45 minutes).	Anytime during weeks 11 to 12
(11) Optional check in session 1	Check in on any technical issues and study logistics with the participant (approximately 15 to 20 minutes).	Anytime during the study and as needed by the participant
(12) Optional check in session 2	Check in on any technical issues and study logistics with the participant (approximately 15 to 20 minutes).	Anytime during the study and as needed by the participant

Intervention participants received 1 daily reminder through CleverCap’s programmed alarm to take their medication at their chosen time. Additional alarms could be programmed by participants, or mobile or app alerts could be set for missed or off-schedule doses, in which case, participants received >1 daily notification. Furthermore, participants were notified if they reopened the pill bottle after an alarm had already been triggered (flagged as an off-schedule dose) or if the cap was improperly closed. However, no reminders were sent, and no alarms were triggered if the CleverCap pill bottle lost power, was deactivated by the study team, was damaged, or lacked a set dosing schedule.

**Figure 1:**
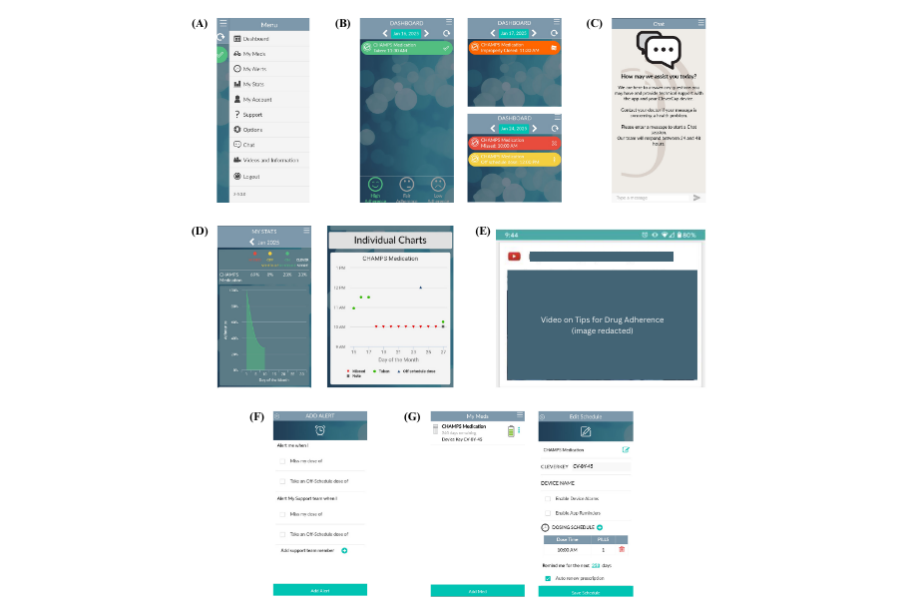
User interface of WiseApp showcasing demo account data: (A) main menu, (B) dashboard with medication adherence status, (C) chat interface, (D) My Stats tab, (E) Videos and Information tab, (F) My Alerts tab, and (G) My Meds tab and Edit Schedule interface.

Figure 1 illustrates the WiseApp user interface, with screenshots of a demonstration account. The main menu (Figure 1A) allows navigation to various tabs. The dashboard (Figure 1B) provides an overview of medication adherence, including statuses such as taken (green), missed (red), off-schedule dose (yellow), and improperly closed cap (orange). It also displays adherence feedback through emoji indicators (eg, high adherence: green smiley face). The chat interface (Figure 1C) enables communication with CHWs, while the My Stats tab (Figure 1D) presents a percentage breakdown of adherence metrics, dose timing, and adherence scores. The Videos and Information tab (Figure 1E) includes testimonial videos with adherence tips. The My Alerts tab (Figure 1F) allows participants and CHWs to set reminders for missed or off-schedule doses. Finally, the My Meds tab (Figure 1G) enables participants and CHWs to manage medication details and adjust dosing schedules.

**Figure 2:**
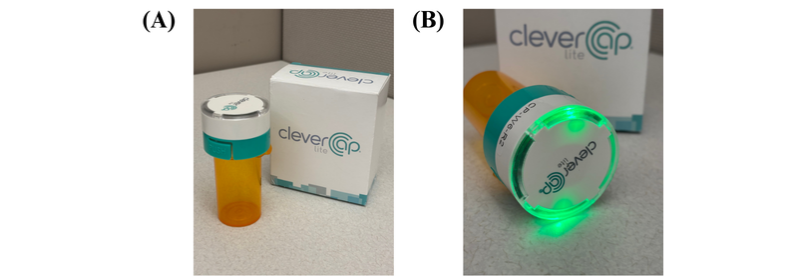
The CleverCap pill bottle used for medication adherence monitoring: (A) device and packaging and (B) active CleverCap alarm for dosing reminder.

The CleverCap pill bottle (Figure 2) tracked medication adherence by recording when the bottle was opened, with a dose marked as “taken” only if the cap remained off for at least 5 seconds. Figure 2A shows the CleverCap and its packaging, which includes a micro-USB charger. Figure 2B demonstrates CleverCap’s built-in reminder system, which provided a visual and auditory alarm when it was time to take a scheduled dose. The bottle flashed green lights for 2 minutes and emitted a loud alarm to ensure that the reminder was noticeable. These cues automatically ceased once the cap was unscrewed, signaling a recorded dose. In addition, if no action was taken within the 2-minute reminder window, the visual and auditory cues stopped, and the dose was recorded as “missed.” No visual or auditory cues were provided for off-schedule doses. If the alarm failed to activate due to incorrect or missing programmed alerts on the WiseApp or due to the pill bottle losing power, participants may have missed their medication without documentation in the app.

### Standard of Care Control Arm

The control arm did not receive any additional interventions beyond the usual standard of care, which consisted of routine clinical services as needed, including referrals to mental health or other ancillary services if clinically indicated.

### Recruitment and Eligibility

Participants were eligible to participate if they (1) were able to speak, read, and write in English; (2) were aged ≥18 years; (3) were willing to provide a valid form of identification for verification; (4) were willing to participate in any assigned arm of the intervention; (5) were diagnosed with HIV ≥6 months ago; (6) had an HIV-1 RNA level of >50 copies/ml or self-reported being virally unsuppressed in the past 12 months (defined as a viral load of ≥200 copies/ml in alignment with the clinical definition of viral suppression in the United States); (7) were owners of a smartphone; (8) were capable and willing to provide informed consent for study participation and consent for access to medical records; and (9) lived in the United States. The use of different viral load thresholds aligns with established clinical guidelines and reflects advances in monitoring technology. A threshold of ≥200 copies/ml is used to determine whether someone is detectable for HIV, as it accounts for small fluctuations in viral levels and testing variations, providing a practical measure of treatment effectiveness [38]. However, with improvements in viral load assay sensitivity, a threshold of 50 copies/ml is increasingly used to identify low levels of HIV in the blood, as achieving a viral load below this threshold is considered optimal for reducing transmission risk and improving long-term health outcomes [39]. Viral load data were obtained through one of the following methods: (1) participant-uploaded viral load test results from their electronic health record or laboratory reports, (2) dried blood spot (DBS) collection analyzed by a certified laboratory, or (3) retrieval through electronic health record access via a HIPAA electronic release of information (eROI) authorization). To ensure consistency in timing, only uploaded viral load results dated within 4 to 6 weeks before the study visit were accepted. DBS collection was offered to all participants as an alternative to laboratory-based testing. Participants were not eligible if they met any exclusion criteria, including (1) residing in a nursing home, prison, or receiving in-patient psychiatric care at the time of enrollment; (2) terminal illness with a life expectancy <3 months; (3) planning to move out of the United States in the next 3 months; or (4) participating in a study that targets viral suppression for people with HIV. The study team carefully considered including newly diagnosed participants (diagnosed <6 months) and chose not to include this subset of people with HIV because newly diagnosed individuals are often treatment naive; thus, their adherence behaviors are unknown and may change frequently as they begin their ART regimen.

Participants were recruited via online advertisements posted on POZ.com, Craigslist, as well as Facebook and Instagram (Meta Platforms, Inc). In addition, the study team emailed community-based organizations serving people with HIV across the country with requests to hang study flyers. To determine preliminary eligibility, participants either filled out a web-based screener or were screened by phone. Participants who filled out web-based screeners were followed up with phone calls and were given detailed instructions regarding the study before scheduling study visits. To finalize eligibility, participants were requested to provide confirmation documentation of viral load either via filling out an eROI, by uploading results obtained within the past 4 to 6 weeks in their electronic health record to a secure study platform, or by participating in a screening visit where trained study staff instructed participants on how to self-collect blood samples via a finger-prick method.

### Study Visits and Visit Structure

#### Overview

The study consisted of 2 required visits: a baseline visit and a 3-month follow-up visit. The study also consisted of an optional screening visit for participants who could not provide confirmation of viral load for eligibility. All study visits were conducted using secure HIPAA-compliant Zoom technology. At the start of each study visit, participants were required to show a valid form of identification for verification and to avert fraud.

#### Screening Visit

Screening visits lasted approximately 1 hour. Participants provided informed consent electronically through the REDCap (Research Electronic Data Capture; Vanderbilt University) system, a secure platform for data collection [40,41]. Before consenting, participants had the opportunity to ask study staff any questions about the process. Before the screening visit, participants were sent a DBS collection kit along with detailed instructions. Trained study staff guided participants through the DBS collection process, which included the use of a Becton Dickinson Microtainer contact-activated lancet blade (1.5 mm × 2.0 mm; blue, high flow) to collect the sample. Care instructions for the puncture site were provided to ensure proper aftercare. Participants were instructed on how to package and ship their samples back to the laboratory at the Miriam Hospital in Rhode Island for processing. Upon receipt of laboratory results, study staff followed up with the participants to inform them of their eligibility for the study. Compensation was not provided for screening visits.

#### Baseline Visit

Baseline visits lasted 2 hours. Participants provided e-consent through REDCap, with the opportunity to ask study staff any questions before participating in the baseline visit. Upon enrollment, participants were randomized to either the intervention or control arm through a randomization module in REDCap. The randomization module was generated by the study team’s data manager, ensuring an unbiased allocation process, while study staff, who were separate from the data manager and did not have access to the randomization module, were responsible for assigning participants to their respective study groups. Intervention participants were informed that they would be sent a CleverCap bottle and would have up to 12 study sessions over the duration of the study with an assigned CHW. Participants were required to complete a comprehensive survey via Qualtrics (Qualtrics International Inc). The survey gathered information on demographics, a single-item ART adherence self-reported scale item (SRSI) [42], and self-efficacy as a mediator of HIV treatment adherence (HIV Treatment Adherence Self-Efficacy Scale [HIV-ASES]) [43]. Baseline viral load and CD4 counts were recorded from information obtained via eROI, participant self-uploaded laboratory results, or the screening visit. Participants were compensated US $40 in the form of an Amazon gift code for their time.

#### 3-Month Follow-Up Visit

Follow-up visits lasted 1 hour. Participants completed a similar survey as the one administered at baseline with the addition of measures for usability displayed to intervention participants, including the Health Information Technology Usability Evaluation Scale (Health-ITUES) [44] and the Poststudy System Usability Questionnaire (PSSUQ) [45]. Participants also provided viral load and CD4 counts obtained via eROI, participant self-uploaded laboratory results, or DBS collection. Real-time adherence data from the CleverCap pill bottles were downloaded from the CleverCap website. At the end of the follow-up visit, all participants were given the option to participate in an optional qualitative interview regarding their experience in the study and the perceived utility of the remotely delivered CHAMPS intervention. Follow-up interviews were conducted exclusively using secure HIPAA-compliant Zoom technology and were audio recorded for transcription. Consent for follow-up interviews was included in the study consent offered to participants at the baseline visit. Participants were compensated US $50 for their time in the follow-up visit and an additional US $35 if they opted to participate in the interview. Compensation was provided in the form of Amazon gift codes.

### Quantitative Data Analysis

Descriptive statistics for demographic variables were reported as counts and percentages. Statistical significance for differences between the control and intervention groups was assessed using the Mann-Whitney *U* test for continuous variables, such as age, and the chi-square test for the remaining categorical variables. A 2-sample *z* test for equality of proportions, with a continuity correction, was used to assess whether the differences in retention rates between the intervention and control groups were statistically significant.

The primary outcome, viral load (virally unsuppressed vs suppressed), was analyzed using a 2-way frequency table by time. The adjusted odds ratio (aOR), adjusting for baseline viral load status, was calculated for the CHAMPS group (intervention vs control).

Self-rated adherence scores using the SRSI [42], a 6-point Likert scale ranging from 1 (“very poor”) to 6 (“excellent”), were reported as mean and SD. Independent 2-tailed *t* tests were used to compare self-rated adherence scores between the intervention and control groups, while paired 2-tailed *t* tests were used to compare scores within groups over time. Missing data for the paired 2-tailed *t* test were handled under the assumption that they was missing completely at random, as participants were lost to follow-up due to external factors unrelated to study data collection.

For adherence data collected through CleverCap, the average percentage of doses taken and missed was calculated, with SD reported for the participants in the analyzed intervention group. The correlation between follow-up SRSI scores and the average percentage of doses taken was calculated using Pearson correlation.

Usability measures, including the PSSUQ [45] and the Health-ITUES [44], were analyzed by calculating the mean and SD for the overall scales and their subscales. The PSSUQ (18 items and 3 subscales) was measured using a 7-point Likert scale, ranging from 1 (“strongly agree”) to 7 (“strongly disagree”). The Health-ITUES (20 items and 4 subscales) was measured using a 5-point Likert scale, ranging from 1 (“strongly disagree”) to 5 (“strongly agree”).

Self-efficacy for HIV treatment adherence was measured using the HIV-ASES (12 items and 2 subscales) [43], a 10-point Likert scale ranging from 1 (“cannot do at all”) to 10 (“certain can do it”). Mean and SD were computed for self-efficacy scores. Independent 2-tailed *t* tests were used to compare HIV-ASES scores between groups, while paired 2-tailed *t* tests were used to compare scores within groups over time, with missing data handled as missing completely at random due to external factors unrelated to the study.

Cronbach α was calculated for scales such as SRSI, Health-ITUES, PSSUQ, and HIV-ASES to measure internal reliability. Statistical analyses were conducted using SAS (SAS Institute) [46] and R software (R Foundation for Statistical Computing) [47], and significance was determined at a *P* value of .05.

### Qualitative Data Analysis

**Figure 3:**
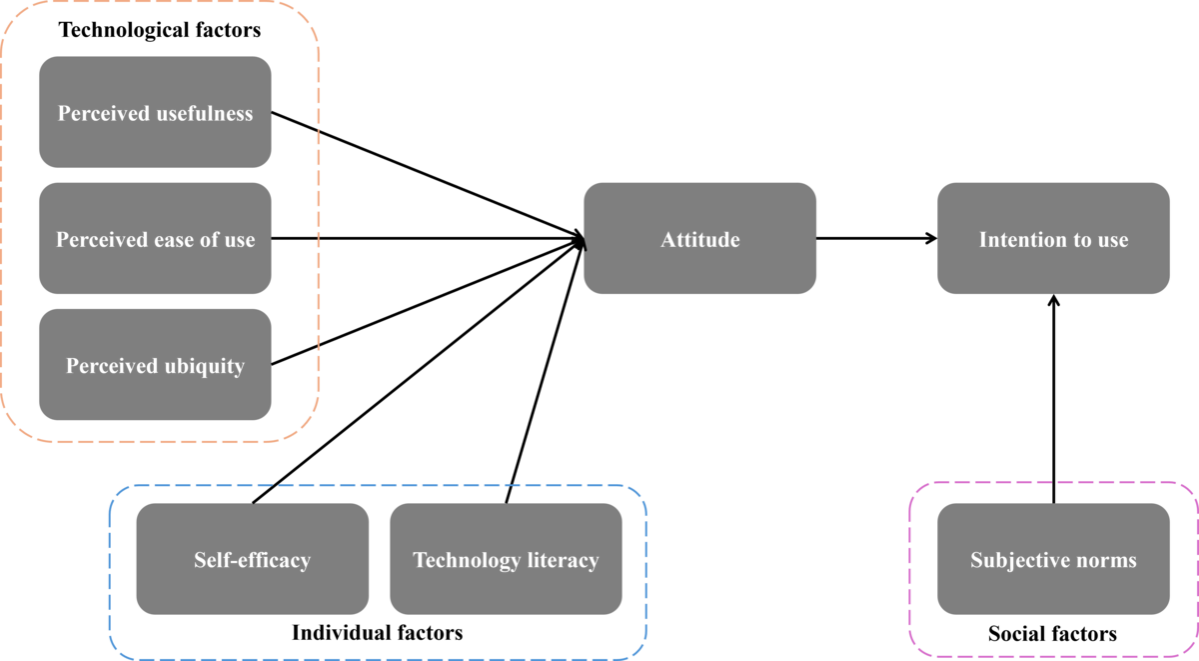
The Mobile Health Technology Acceptance Model adapted to analyze participant perceptions of the Community Health Worker and Mobile Health to Improve Viral Suppression (CHAMPS) intervention and experiences participating in the CHAMPS pilot study.

Following transcription, 3 members of the research team (SS, SM, and TC) conducted a double-coding process on the transcripts from 52% (12/23) intervention group participants and 48% (11/23) control group participants. An initial codebook was developed based on the Mobile Health Technology Acceptance Model (MHTAM), which includes technological, individual, and social factors that influence the acceptance of health care technology [22]. This model has been adapted for this study to examine an individual’s intention to use mHealth interventions for HIV treatment adherence or participate in the CHAMPS pilot study procedures, as it examines key factors such as perceived usefulness, ease of use, and ubiquity, alongside individual beliefs and social influences (Figure 3). Data analysis was conducted using Excel (Microsoft Corporation), which has been demonstrated as a practical tool for qualitative analysis due to its capabilities for organizing, coding, and analyzing textual data [48]. The methodological approach was guided by Ose [49], who outlined systematic steps for using Excel to manage and code qualitative data efficiently.

The codebook was created to align with the themes from the interview guide, resulting in the following finalized codes: (1) perceived usefulness, (2) perceived ease of use, (3) perceived ubiquity (ie, the perception of seamless access to health care networks and services at any time and place), (4) self-efficacy, (5) technology literacy, and (6) subjective norms. In addition, the codebook was applied deductively to analyze barriers and facilitators related to both the CHAMPS intervention and study procedures.

The coding process involved all coders coding 2 transcripts for initial consensus with the codebook and double coding 81% (17/21) of the remaining transcripts to enhance reliability, with regular meetings to compare and synthesize definitions for consistency. In certain cases, a consensus coder resolved discrepancies to ensure agreement. A small number of transcripts (4/21, 19%) were single coded due to availability constraints. Overall, double coding and consensus coding were applied to improve intercoder reliability and ensure a rigorous analytic process [50,51].

### Ethical Considerations

All study procedures were reviewed and approved by the Columbia University Institutional Review Board (protocol number AAAU2064) before the recruitment or enrollment of participants. Participants provided electronic written consent, including screening consent for those requiring a screening visit and study consent for those proceeding directly to baseline. The baseline consent included language informing participants about an optional follow-up interview, with participants indicating their willingness to participate by initialing the consent form. Study data, including interview transcripts, were anonymized and deidentified to ensure confidentiality. Participants were compensated as follows: no compensation for the screening visit, US $40 for the baseline visit, US $50 for the follow-up visit, and US $35 for the optional qualitative interview at the follow-up visit. All images and data presented in this manuscript are deidentified to ensure the privacy of our participants.

## Results

### Participant Characteristics

#### Overview

**Figure 4:**
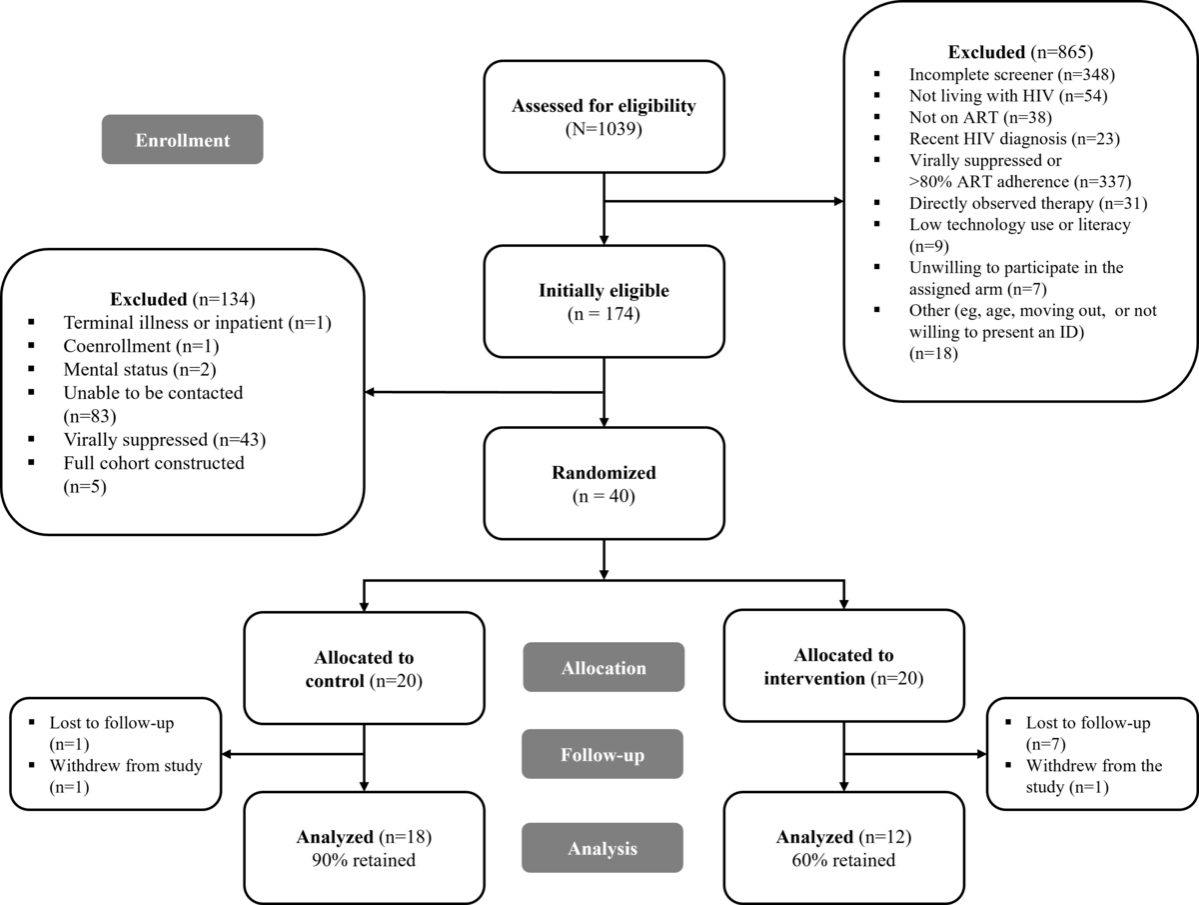
CONSORT (Consolidated Standards of Reporting Trials) flow diagram of participant enrollment, allocation, follow-up, and analysis within the Community Health Worker and Mobile Health to Improve Viral Suppression (CHAMPS) pilot study.

A total of 40 participants were enrolled in the study, with 20 (50%) participants each randomly assigned to the intervention and control groups. Retention rates were lower in the intervention group (12/20, 60%) compared to the control group (18/20, 90%). Although retention was higher in the control group, this difference was not statistically significant (*P*=.07; Figure 4). Overall, the study retained 75% (30/40) of the participants, reflecting strong follow-up rates and participation in the study.

#### Participant Demographics

At baseline, participant demographics were well balanced between the intervention and control groups, indicating that randomization was effective. There were no statistically significant differences in key sociodemographic variables, including age, gender, race, or income level, between the 2 groups (all *P*>.05). The median age of the participants in the control group was 51.5 (IQR 22.0) years, compared to 45.0 (IQR 16.8) years in the intervention group. Gender distribution was similar, with 60% (12/20) of participants in each group identifying as male and 40% (8/20) of participants in the intervention group and 35% (7/20) of the participants in the control group identifying as female. 1 participant in the control group (1/20, 5%) identified as “something else” and were provided the opportunity to elaborate more on how they conceptualized their gender. The racial composition was predominantly African American or Black (10/20, 50% in the control group and 14/20, 70% in the intervention group.)

Educational attainment, employment status, and health insurance coverage were also comparable between the groups, supporting the conclusion that randomization successfully minimized baseline differences between the intervention and control arms. Notably, most participants (25/40, 63%) in both intervention and control groups reported annual incomes <US $20,000, potentially highlighting shared socioeconomic barriers that might limit their ability to fully engage with HIV care and maximize its potential benefits (Table 2).

**Table 2:** Baseline sociodemographic characteristics of participants, comparing the control and intervention groups (N=40).

Category		Control (n=20)	Intervention (n=20)	*P* value
Age (y), median (IQR)		51.5 (22.0)	45.0 (16.8)	.53^a^
**Gender, n (%)**				
	Male	12 (60)	12 (60)	.99
	Female	7 (35)	8 (40)	.78
	Something else	1 (5)	0 (0)	.32
**Sex, n (%)**				
	Male	13 (65)	12 (60)	.84
	Female	7 (35)	8 (40)	.80
**Sexual orientation, n (%)**				
	Homosexual, gay, or lesbian	8 (40)	10 (50)	.64
	Heterosexual or straight	9 (45)	7 (35)	.62
	Bisexual	2 (10)	2 (10)	.99
	Asexual	1 (5)	0 (0)	.32
	Something else (please specify)	0 (0)	1 (5)	.32
**Race, n (%)**				
	American Indian, Alaska Native, Native Hawaiian, or other Pacific Islander	0 (0)	0 (0)	.99
	Asian or Asian American	1 (5)	0 (0)	.32
	Black or African American	10 (50)	14 (70)	.41
	White	9 (45)	5 (25)	.29
	Something else	0 (0)	1 (5)	.32
**Ethnicity, n (%)**				
	Hispanic or Latino	2 (10)	2 (10)	.99
	Not Hispanic or Latino	18 (90)	18 (90)	.99
**Relationship status, n (%)**				
	Single	10 (50)	14 (70)	.41
	In a relationship with a man	6 (30)	3 (15)	.32
	In a relationship with a woman	1 (5)	0 (0)	.32
	Divorced or separated from a man	1 (5)	0 (0)	.32
	Divorced or separated from a woman	1 (5)	0 (0)	.32
	Widowed	0 (0)	1 (5)	.32
	Other	1 (5)	2 (10)	.56
**Children, n (%)**				
	No	12 (60)	15 (75)	.56
	Yes	8 (40)	5 (25)	.41
**Education, n (%)**				
	None	0 (0)	0 (0)	.99
	Elementary school	0 (0)	0 (0)	.99
	Some high school or no diploma	2 (10)	3 (15)	.66
	High-school diploma or equivalent (eg, GED^b^)	5 (25)	5 (25)	.99
	Some college	6 (30)	4 (20)	.53
	Associate degree or technical degree	4 (20)	3 (15)	.71
	Bachelor or college degree	2 (10)	3 (15)	.66
	Professional or graduate degree	1 (5)	2 (10)	.56
**Employment status, n (%)**				
	Working full time	3 (15)	4 (20)	.71
	Working part time (eg, seasonal and work-study)	6 (30)	3 (15)	.32
	Unemployed and looking for work	3 (15)	5 (25)	.48
	Unemployed and not looking for work	0 (0)	2 (10)	.16
	Retired	5 (25)	2 (10)	.26
	Disabled	4 (20)	6 (30)	.53
**Annual income (US $), n (%)**				
	<10,000	7 (35)	8 (40)	.80
	10,000-19,999	5 (25)	5 (25)	.99
	20,000-39,999	4 (20)	2 (10)	.41
	40,000-59,999	3 (15)	1 (5)	.32
	60,000-79,999	0 (0)	1 (5)	.32
	80,000-99,999	0 (0)	1 (5)	.32
	100,000-149,999	0 (0)	1 (5)	.32
	≥150,000	1 (5)	0 (0)	.32
	I do not know	0 (0)	1 (5)	.32
**Health insurance status, n (%)**				
	No	1 (5)	0 (0)	.32
	Yes, through my job	0 (0)	1 (5)	.32
	Yes, through a health exchange (Affordable Care Act)	1 (5)	2 (10)	.56
	Yes, Medicaid or Medicare	17 (85)	17 (85)	.99
	Yes, AIDS Drug Assistance Program	3 (15)	3 (15)	.99
	Other	1 (5)	1 (5)	.99
	I do not know	0 (0)	0 (0)	.99

^a^The *P* value for age was calculated using the Mann-Whitney *U* test to compare the distribution of ages between the control and intervention groups based on the full dataset of individual ages.

^b^GED: General Educational Development.

#### Self-Efficacy

**Figure 5:**
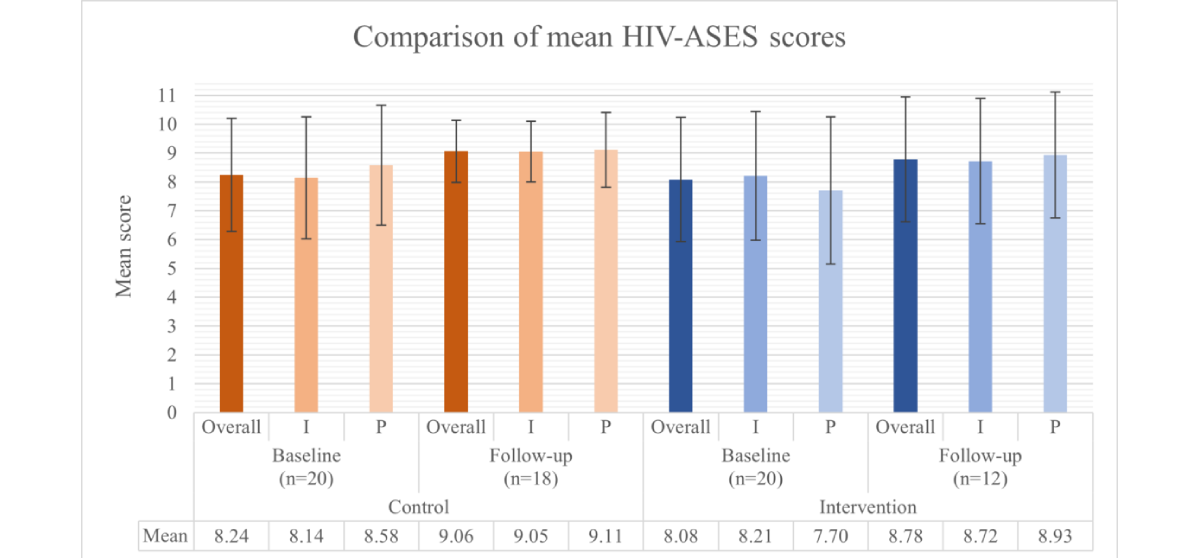
Self-efficacy for antiretroviral therapy adherence among control and intervention group participants, as measured by the HIV Treatment Adherence Self-Efficacy Scale (HIV-ASES) at baseline and follow-up. Error bars represent SDs. No statistically significant differences were observed between groups or within groups over time. I: Integration subscale; P: Perseverance subscale.

Self-efficacy has been recognized as a key factor influencing medication adherence in HIV treatment and other medical conditions [43]. As such, it was quantitatively measured in this study using the HIV-ASES, which has a maximum mean score of 10. At baseline, the control group (20/20, 100%) had a mean HIV-ASES overall score of 8.24 (SD 1.96), and the intervention group (20/20, 100%) had a mean score of 8.08 (SD 2.25). At follow-up, the control group’s (18/20, 90%) mean score increased to 9.06 (SD 1.08), while the intervention group’s (12/20, 60%) mean score was 8.78 (SD 2.16). Although unexpected, the intervention group’s lower self-efficacy score compared to the control group may reflect increased awareness of their adherence habits through engagement with the CHAMPS intervention, leading to more accurate and self-critical reporting. Conversely, social desirability bias may have inflated the control group’s self-reported scores. No significant differences were observed between the groups at baseline (*P*=.81) or follow-up (*P*=.64), and no significant within-group changes were observed over time (control: *P*=.52 and intervention: *P*=.07; Figure 5). The overall scale had excellent internal consistency, with Cronbach α=0.95 at baseline and Cronbach α=0.98 at follow-up [52].

For the integration subscale, which includes the first 9 questions on the HIV-ASES and measures participants’ ability to incorporate treatment into their daily lives [43], mean baseline scores were 8.14 (SD 2.11) for the control group and 8.21 (SD 2.23) for the intervention group. At follow-up, the mean scores increased to 9.05 (SD 1.05) and 8.72 (SD 2.17), respectively. No significant between-group differences were found at baseline (*P*=.92) or follow-up (*P*=.58), and there were no significant within-group changes (control: *P*=.26 and intervention: *P*=.89; Figure 5). The integration subscale had excellent reliability, with Cronbach α=0.95 at baseline and Cronbach α=0.97 at follow-up [52].

For the perseverance subscale, which includes the final 3 questions on the HIV-ASES and measures participants’ confidence in maintaining adherence to their treatment regimens despite any challenges [43], the control group’s baseline mean score was 8.58 (SD 2.08), and the intervention group’s mean score was 7.70 (SD 2.55). Follow-up mean scores were 9.11 (SD 1.30) for the control group and 8.93 (SD 2.18) for the intervention group. No significant between-group differences were found at baseline (*P*=.24) or follow-up (*P*=.78), and no significant within-group changes were observed (control: *P*=.81 and intervention: *P*=.32; Figure 5). The perseverance subscale had acceptable and good reliability, with Cronbach α=0.87 at baseline and Cronbach α=0.93 at follow-up [52].

The quantitative findings, which indicated high self-efficacy in both the control and intervention groups, were further supported by qualitative feedback from participants, who described their personal commitment to staying on top of their medication routines. 1 participant shared how self-efficacy played a role in maintaining adherence:

Because I just like to have a good track record of me taking my medication in a timely fashion. So, when I saw that face wasn’t a smile, and it was a frown, it made me stay on top of my, you know, on my game in terms of me being more consistent. Well, maintaining my consistency with taking my medication.

CHP 13

These findings highlight the strong sense of personal responsibility and self-efficacy that participants expressed in their medication adherence, where they were motivated by a desire to maintain their own health. A detailed list of additional illustrative quotes organized by the MHTAM themes and their respective definitions is provided in Multimedia Appendix 1.

### Delivered Intervention

A total of 10 CHW sessions were conducted as planned, although the optional 2 additional sessions were not used due to declining participant engagement and the proximity of some sessions to the participants’ final follow-up visits. CHWs used HIPAA-compliant Zoom calls, phone calls, and the WiseApp chat feature based on participant preferences. Completion rates (Table 3) were initially high, with 17 (85%) out of the 20 intervention participants completing sessions 1 to 3, but this declined to 9 (45%) participants by the 10th session. Participants generally preferred phone calls for session delivery, with a consistent majority opting for this modality across all sessions. The variability in delivery modes during sessions 1 and 2 reflects logistical factors: some participants had not received their CleverCap devices at the time of their baseline visit, necessitating follow-up sessions via Zoom call or phone call for session 2. In addition, 3 participants disengaged from the study immediately after the baseline visit, which contributed to the drop in session completion rates early in the intervention. To re-engage participants who missed sessions, CHWs attempted up to 3 follow-ups using participants’ disclosed communication preferences (calls, SMS text messaging, or emails). When no response was received after 3 attempts, re-engagement efforts were discontinued.

**Table 3:** Completion rates and delivery model for community health worker sessions among participants in the intervention arm (n=20).

Session number	Participants completing the session, n (%)	Mode of session delivery, n (%)		
		Zoom	Phone	WiseApp chat
1	17 (85)	9 (53)	8 (47)	0 (0)
2	17 (85)	3 (18)	14 (82)	0 (0)
3	17 (85)	1 (6)	15 (88)	1 (6)
4	13 (65)	0 ()	13 (100)	0 (0)
5	13 (65)	1 (8)	11 (85)	1 (8)
6	13 (65)	0 (0)	13 (100)	0 (0)
7	13 (65)	0 (0)	12 (92)	1 (8)
8	12 (60)	1 (8)	11 (92)	0 (0)
9	12 (60)	1 (8)	10 (83)	1 (8)
10	9 (45)	2 (22)	7 (78)	0 (0)

The CleverCap pill bottles issued 3151 notifications, comprising notifications for taking their medication and alerts for nonadherence or improper use (Table 4). Notifications sent per participant varied widely, with a mean value of 158.3 (SD 99.9). Participants receiving >200 notifications often had frequently missed or off-schedule doses, while those receiving <90 notifications likely disengaged from the intervention by deactivating, losing, or destroying their pill bottles. This 90-notification threshold was based on the intervention design, which included 1 scheduled daily reminder over the 3-month study period (3 months ×30 days=90 reminders per participant). Any participant receiving substantially <90 notifications likely did not use the device consistently, whereas those receiving >90 notifications frequently triggered additional alerts due to missed or off-schedule doses.

**Table 4:** Frequency and distribution of medication adherence notifications issued by the CleverCap pill bottle to participants in the intervention arm (N=3151).

Notification type	Notifications, n (%)
Dose taken	1061 (33.67)
Missed dose	1585 (50.3)
Off-schedule dose	418 (13.26)
Improperly closed pill bottle	87 (2.76)

### Intervention Characteristics

#### Overview

The aOR for achieving undetectable viral load in the intervention group compared to the control group was 3.01 (95% CI –1.59 to 4.12), controlling for baseline viral load status (Table 5). This aOR represents a medium effect size, suggesting an improvement in viral suppression in the intervention group [53]. Baseline viral load status by follow-up status and group is presented in Multimedia Appendix 2.

**Table 5:** Baseline and follow-up viral load status of control and intervention group participants (N=40).

Viral load^a^	Baseline, n (%)				Follow-up, n (%)		
	Control (n=20)	Intervention (n=20)	Total (n=40)	Control (n=18)		Intervention (n=12)	Total (n=29)
Not detectable (<200 copies/ml)	17 (85)	11 (55)	28 (70)	15^b^ (83)		11 (92)	26 (90)
Detectable (≥200 copies/ml)	3 (15)	9 (45)	12 (30)	2 (11)		1 (8)	3 (10)

^a^For consistency with US clinical guidelines, viral load status is categorized using a threshold of ≥200 copies/ml to define detectability. While participants with viral loads between 50 and 199 copies/ml were eligible for the study, they were classified as suppressed in this analysis. In addition, details on the methods used to obtain viral load measurements at baseline and follow-up are provided in Multimedia Appendix 3.

^b^Data missing (1/18, 6%) as an insufficient sample provided by 1 participant precluded analysis and quantification.

Notably, nearly three-fourths of the participants (28/40, 70%) had an undetectable viral load (<200 copies/ml) at baseline. Among those with undetectable viral load at baseline, viral suppression was largely maintained throughout the study period. Specifically, in the control group, the viral load of 1 participant became detectable at follow-up, whereas in the intervention group, all participants’ viral loads that were initially undetectable remained undetectable. Among participants with a detectable viral load (≥200 copies/ml) at baseline, 44% (4/9) of individuals in the intervention group achieved viral suppression by follow-up, compared to only 33% (1/3) in the control group.

**Figure 6:**
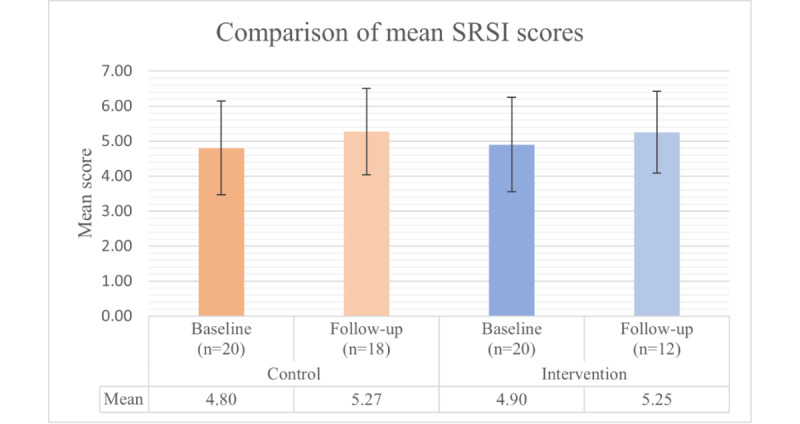
Changes in self-reported adherence to antiretroviral therapy among control and intervention group participants, as measured using the self-reported scale item (SRSI) at baseline and follow-up. Error bars represent SDs. No statistically significant differences were observed between groups or within groups over time.

At baseline, the control group (20/20, 100%) had a mean SRSI score of 4.80 (SD 1.34), while the intervention group (20/20, 100%) had a mean score of 4.90 (SD 1.35). At follow-up, the control group’s (18/20, 90%) mean increased to 5.27 (SD 1.23), and the intervention group’s (12/20, 60%) mean was 5.25 (SD 1.17). No significant differences were observed between the groups at baseline (*P*=.77) or follow-up (*P*=.95), and no significant within-group changes were observed over time (control: *P*=.16 and intervention: *P*=.29; Figure 6). The SRSI score showed excellent internal consistency, with Cronbach α=0.96 at baseline and Cronbach α=0.98 at follow-up [52].

Adherence data from CleverCap showed that intervention group participants (12/20, 60%) took an average of 60.08% (SD 19.15%) of their prescribed doses, with 39.33% (SD 19.24%) of doses missed. A weak, nonsignificant correlation was found between the CleverCap adherence data (percentage taken) and follow-up SRSI scores (*r*=0.121; *P*=.71), suggesting that the high self-reported adherence scores did not strongly align with the electronic monitoring data.

Participant feedback can help explain the discrepancy between self-reported scores and actual adherence by highlighting individual factors, such as personal medication management preferences, that influence adherence. 1 participant noted the following, illustrating how participants’ personal beliefs about the medication’s dosing flexibility influenced their adherence behavior:

I know that Biktarvy has a 50-hour shelf-life. And so, I’m not really stressing over when I take it. I usually just make sure I take it before lunch.

CHP 2

Rather than relying on CleverCap’s programmed alarms, participants adjusted their dosing schedules based on their own understanding of the medication’s efficacy, which may have contributed to lower adherence rates recorded by the pill bottle.

#### Feasibility of the Remotely Delivered CleverCap Pill Bottle and the WiseApp Intervention

The feasibility of the intervention was assessed by supplementing usability data with feedback regarding participants’ attitudes and intentions to use the intervention [54,55]. The usability of the intervention was assessed using the Health-ITUES and PSSUQ scores, which provided insights into participants’ experiences and satisfaction with the intervention, respectively. The Health-ITUES had a maximum mean score of 5, with higher scores indicating better usability. Participants (12/20, 60%) rated the overall usability of the intervention positively, with an average overall score of 4.35 (SD 0.58). The scores ranged from 3.50 to 5, with an IQR of 0.84, reflecting consistently high usability ratings. Specific subscales, such as perceived usefulness (mean 4.42, SD 0.63) and perceived ease of use (mean 4.47, SD 0.71), received similarly high ratings, with IQRs of 1.11 and 0.50, respectively, indicating that participants generally found the pill bottle and app useful and easy to use (Table 6).

**Table 6:** Usability and acceptability of the Community Health Worker and Mobile Health to Improve Viral Suppression intervention, as assessed by the Health Information Technology Usability Evaluation Scale (Health-ITUES) and Poststudy System Usability Questionnaire (PSSUQ) among intervention group participants.

Scale		Scores, mean (SD)	Scores, median (IQR; range)	Cronbach α
**Health-ITUES (n=12)**				
	Overall	4.35 (0.58)	4.48 (0.84; 3.50-5)	0.94
	Impact	4.36 (0.66)	4.17 (1.08; 3.33-5)	0.92
	Perceived usefulness	4.42 (0.63)	4.33 (1.11; 3.11-5)	0.83
	Perceived ease of use	4.47 (0.71)	4.90 (0.50; 3.67-5)	0.63
	User control	3.86 (0.95)	4.07 (1.75; 2.33-5)	0.79
**PSSUQ (n=12)**				
	Overall	2.04 (1.03)	3.81 (1.31; 1-4.19)	0.96
	System usefulness	1.87 (1.09)	1.79 (0.79; 1-4.86)	0.94
	Information quality	2.13 (1.16)	1.67 (2.04; 1-3.67)	0.96
	Interface quality	2.05 (0.98)	2.17 (1.58; 1-4.33)	0.86

These high ratings are echoed in participant feedback. The pill bottle’s visual presence was perceived as useful for maintaining adherence. Participants also found the pill bottle easy to operate and intuitive, sharing positive experiences as follows:

Because I’m not really good with taking it at a certain time every day, at the same time every day. And so I feel like the CleverCap helped me with that...It does remind me, because I always see it, versus me keeping it in a white bottle. When I see the CleverCap on my dresser, it just reminds me. “Okay, it’s time to take your medicine.”...The functions of the CleverCap were easy to use. I didn’t have to charge it many times. I charged it once. It’s very easy. It reminded me to take my medicine. I really, really appreciated that. I just love it. It’s very easy. And it definitely reminded me to stay on my meds.

CHP 39

However, the user control subscale received a lower average score (mean 3.86, SD 0.95) and a wider IQR of 1.75, suggesting more variability in responses and some limitations in how participants felt they could manage the intervention. These concerns were reflected in feedback about the pill bottle’s fragility and usability during travel:

Because there were times I was out of town, and I don’t like bringing the pill bottle with me...I didn’t take the bottle. I took my medication, but I didn’t take the reminder, the bottle...That container, with the alarm on, it’s very sensitive. I dropped it one time; I was afraid it might break...So, that’s why when I knew I was leaving to go out of town, I just felt it was more secure leaving it at home.

CHP 13

Despite this, some participants (3/12, 25%) were able to maintain adherence even when separated from the pill bottle, relying on their own strategies for medication management. 1 participant reflected on this experience, demonstrating that while CleverCap supported adherence, participants’ self-efficacy and personal management strategies often played a crucial role in overcoming barriers related to the pill bottle’s portability and user control:

When I went to D.C., I forgot the CleverCap...But I had my medicine. I just got the notifications like, oh, it’s time to take your medicine. Didn’t bring the cap.

CHP 32

The PSSUQ had a maximum mean score of 7, where lower scores indicate better usability. Participants reported generally high satisfaction with the system’s usability, with an average overall score of 2.04 (SD 1.03). The scores ranged from 1 to 4.19, with an IQR of 1.31, indicating a favorable user experience with some variability. Ratings for system usefulness (mean 1.87, SD 1.09) and interface quality (mean 2.05, SD 0.98) subscales also indicate positive user experiences, with IQRs of 0.79 and 1.58, respectively, although information quality subscale (mean 2.13, SD 1.16) showed highest variability in participant responses, with an IQR of 2.04 (Table 5).

To assess the internal reliability of the scales used, Cronbach α was calculated for both the Health-ITUES and PSSUQ. The Health-ITUES demonstrated excellent overall reliability (Cronbach α=0.94), with variable consistency across the following subscales: perceived usefulness (Cronbach α=0.92, excellent), integration (Cronbach α=0.83, good), perceived ease of use (Cronbach α=0.63, acceptable), and user control (Cronbach α=0.76, acceptable). The PSSUQ exhibited excellent overall reliability (Cronbach α=0.96), with high consistency across the following subscales: system usefulness (Cronbach α=0.94, excellent), information quality (Cronbach α=0.96, excellent), and interface quality (Cronbach α=0.86, good) [52].

Our results suggest that, despite potential improvements in user control and information clarity, the remote delivery of the CleverCap pill bottle and WiseApp was generally well received, demonstrating feasibility and ease of use.

#### Perceptions of CHW Sessions

Participants shared a variety of experiences regarding their engagement with the CHW sessions, with responses generally falling into 3 key themes: ease of use and self-efficacy as a facilitator; positive social influence; and barriers to ubiquity, such as infrastructure or scheduling challenges.

Several participants (10/12, 83%) expressed that the flexibility and accessibility of the CHW sessions facilitated their engagement. The ability to communicate with CHWs through various channels, such as text, phone, and email, was highly appreciated. For instance, 1 participant highlighted the ease of communication with and persistence of the CHWs. In addition, participants with existing knowledge about HIV felt comfortable discussing their treatment with the CHWs, further facilitating their engagement:

The flexibility that you had was amazing. Like I said, you guys were available through a text message, through a phone call, through email...whatever it took. You guys didn’t disappear. You guys didn’t give up. You guys are like, hello, I am trying to talk to you, and kept on it. So, that was cool...To a person who already knows about HIV, I am completely comfortable talking about anything that has to do with it. So, that wasn’t an issue for me.

CHP 17

Positive social influence, particularly through regular check-ins and motivational support from the CHWs, emerged as a key facilitator of engagement. Participants expressed appreciation for the ongoing support provided by the CHWs, with some (10/12, 83%) noting that the regular communication made them feel more accountable and supported in their adherence:

[The study] really was awesome. I mean the perk was having you call me once a week...Having your own personal somebody to motivate you and check up on you and make sure you're doing good and adhering to your medicine.

CHP 39

While many participants found the CHW sessions helpful, some (2/12, 16%) faced barriers to engagement due to external factors, such as infrastructure limitations or time constraints. Participants living in rural areas with limited internet access found it challenging to maintain consistent communication with CHWs. 1 participant from a rural area explained how poor internet connectivity hindered their participation:

Like I said, I’m busy. The issue with scheduling because, just like I said, I was always on the go. I’m always on the go...Travel, not having internet for interaction with you. That was sometimes challenging, I guess. Where I live, here in rural Alabama, I dare say that 75% of the county... [does] not have any kind of access to internet. It’s scarce...the internet and phone service in a lot of spaces. And so that would be a challenge to speaking with you, as well as trying to look at any data on the phone.

CHP 38

In addition, some participants (4/12, 33%) mentioned that their busy schedules made it difficult to consistently engage in the CHW sessions (Multimedia Appendix 1).

Thus, while there were some challenges related to infrastructure and scheduling, participants’ perceptions suggest that the CHW sessions were generally well received, particularly in terms of flexibility, ease of communication, and motivational support.

### Acceptability of Remote Study Procedures

The acceptability of delivering the intervention and conducting study procedures remotely was assessed through participant feedback, which highlighted the overall usefulness of participating in the study; ease of remote interactions; and logistical considerations, such as shipping and blood sample collection.

Participants largely embraced the flexibility offered by remote study visits conducted via Zoom. Many (15/23, 65%) expressed comfort and appreciation for the convenience of participating from their own homes, along with increased privacy and adaptability. 1 participant noted the following:

I have used Zoom in the past for like work...But with Zoom, it’s actually more...what’s the word I’m looking for? This was more private...If I had to step out and go somewhere, I still could do it that way. But, I felt more comfortable doing it on my laptop, you know, at home. And, I liked the flexibility I had with you all, as well. So, it was great, because you all worked around my schedule.

CHP 23

Overall, participants found Zoom easy to use and appreciated the accessibility of conducting study visits remotely, especially in cases where mobility or health concerns made in-person visits difficult. 1 participant explained as follows:

Doesn’t bother me. I’d rather [Zoom] than have to come out. It’s difficult for me to walk right now...This is good because at least I can participate in things. And I don’t have to make myself uncomfortable to do it, you know?

CHP 30

Remote collection of blood samples, facilitated through mailed kits, was another aspect of the study that participants generally found convenient. 1 participant described the process as efficient and accessible:

I think [the blood sample collection process is] very convenient. I’m nowhere near you guys and normally, you know, we’d have someone say, well, you’re not able to come into the office and do the blood draws or whatever. So, this was very convenient.

CHP 11

Furthermore, participants expressed appreciation for the comprehensive kits and clear instructions provided (Multimedia Appendix 1). Although some challenges with blood sample collection were reported, including minor issues such as bruising and difficulties with blood flow, these were anticipated and communicated during the informed consent process, as such challenges are commonly associated with point-of-care testing [56]. Multimedia Appendix 3 provides additional numeric data supporting these findings.

The shipping process for study kits and the CleverCap pill bottle was largely seamless for participants, who noted the discreet packaging of materials as a positive aspect of the study. 1 participant mentioned how the packaging helped protect their privacy:

Just because of the delicateness of the subject matter, it’s essentially discreet because nobody thinks a FedEx package is anything suspect or scary. It’s just a FedEx package...it blended right in and it doesn’t cause any red flags or make anybody feel uncomfortable in a public setting.

CHP 17

However, there were occasional logistical challenges with returning the packages. A few participants (2/23, 9%) noted issues with transportation and scheduling FedEx pickups, which delayed their ability to return the materials. 1 participant shared the following:

Because, I don’t always have transportation, so it’s been harder for me to get the package sent back in like a timely manner. I know you guys have tried setting up a pick-up with FedEx, but they never came to pick up the package. So, that’s like been the only barrier I’ve had in participating in the program and getting the package to a FedEx location.

CHP 27

While logistical challenges, such as transportation for package returns, were noted, the study’s design was practical and effective for most participants, supporting its acceptability and viability in remote settings.

## Discussion

### Principal Findings

This pilot study aimed to primarily assess the feasibility and usability of the remotely delivered CHAMPS intervention. While the preliminary results did not demonstrate significant improvements in adherence or self-efficacy in the intervention group compared to the control group, the findings provide important insights into how participant attitudes to use the intervention were shaped by technological, individual, and social factors. Thus, understanding these sociodemographic factors is essential for contextualizing the intervention’s feasibility and the challenges participants faced in engaging with the CHAMPS intervention. The demographic characteristics of participants in this study partially align with key sociodemographic characteristics of people with HIV in the United States. These parallels suggest that our findings may be applicable to subsets of the broader population of people with HIV, particularly those disproportionately affected by intersecting socioeconomic and racial disparities. Most of our participants (24/40, 60%) identified as Black or African American individuals, consistent with national data showing that nearly half of people with HIV belong to this group [57]. The predominance of participants reporting annual incomes <US $20,000, which is nearly double the proportion of individuals living below the federal poverty level (US $15,060/y) compared to the national estimate of one-third of people with HIV, may reflect competing priorities that made it challenging to fully engage with the intervention and may help explain the nonsignificant results observed [57].

Baseline HIV-ASES scores showed that participants in both control and intervention groups had high self-efficacy, particularly in integrating HIV medication into their routines and maintaining long-term adherence. This is critical, as self-efficacy is a well-established predictor of successful health behavior change, particularly for chronic conditions such as HIV [58,59]. Although modest increases in scores were observed at follow-up, they were not statistically significant, and the control group reported higher self-efficacy than the intervention group, indicating that the intervention did not significantly enhance an already strong sense of self-efficacy. Qualitative feedback confirmed that participants relied on their preexisting knowledge and ability to adhere to medication, making additional tools unnecessary.

SRSI scores showed stable adherence in both groups from baseline to follow-up, with slight, nonsignificant increases. Although the intervention did not significantly improve adherence, qualitative data revealed that participants, especially those with higher self-efficacy, often prioritized their own understanding of medication efficacy over CleverCap reminders. High confidence in managing their treatment may have made these reminders less relevant, explaining the lack of significant differences between groups.

Health-ITUES results showed the intervention’s success in usability, with an overall score of 4.35 exceeding the acceptable cut point of 4.32 [60]. High scores in perceived usefulness and ease of use subscale indicated that participants found WiseApp helpful and intuitive, supporting the MHTAM framework’s emphasis on these factors for technology adoption [22]. However, lower scores in user control revealed challenges with CleverCap’s portability, aligning with MHTAM’s perceived ubiquity construct. PSSUQ scores also reflected positive usability, with an overall mean of 2.04 (SD 1.03). Participants rated system usefulness and interface quality highly.

Subjective norms emerged as an important factor for engagement with the CHW component of the intervention. The regular check-ins and motivational support from CHWs were highlighted as valuable by participants, reinforcing the social component of adherence. This reflects the broader role of CHWs in providing not only practical assistance but also emotional support, which has been shown to improve health outcomes in other studies [21-23].

This pilot study found high acceptability for remotely delivered procedures, with participants appreciating the convenience, privacy, and flexibility of remote interactions. Zoom visits were well received, allowing participation from home, which aligns with literature suggesting that remote health care improves patient satisfaction by reducing travel burden and enhancing accessibility [61-63]. These procedures were key in promoting participation and retention. Blood sample collection via mailed kits was convenient for most participants (27/30, 90%), demonstrating that, with clear guidance, it is a feasible alternative to in-person phlebotomy. Discreet packaging of study materials was also valued, as it addressed privacy needs and reduced HIV stigma, which is a critical factor in engaging people with HIV in care [64,65].

Our findings highlight the potential of mHealth interventions, such as CHAMPS, to support the “Treat” pillar of the Ending the HIV Epidemic initiative by enhancing accessibility and convenience for people with HIV. The usability and acceptability of remote delivery methods, such as mobile apps for medication management, CHW support, and mailed DBS collection, demonstrate a promising foundation for addressing barriers to care. These strategies could be further refined to promote viral suppression and improve engagement in HIV care for populations facing challenges in accessing traditional health care services.

### Limitations

This study has several limitations that should be considered. The small sample size of 40 participants limited the statistical power, making it difficult to detect significant differences between groups and reducing the generalizability of findings to the broader populations of people with HIV. Furthermore, the small sample size for positive outcome events, such as viral suppression at follow-up, may have affected the reliability of the effect size estimate, particularly within the intervention group. The lower retention rate in the intervention group compared to the control group, while not statistically significant, suggests challenges in effectively engaging participants assigned to the intervention. This highlights the need to address structural and social determinants of health when designing ART adherence interventions. Future research should explore strategies to reduce these barriers, such as more flexible engagement options or additional resources.

In addition, recruitment challenges, particularly among younger people with HIV (aged 18 to 29 y), were notable. Social media platforms used for advertising, such as POZ.com, primarily attracted older participants due to their demographic reach. Alternative platforms, such as TikTok, which are more commonly used by younger populations, should be prioritized in future campaigns [66]. Strategies such as peer-driven recruitment and culturally tailored SMS text messaging could help engage younger individuals who may be less trusting of traditional health care outreach methods. Addressing time constraints and providing more flexible scheduling options may also increase participation among this demographic [67,68]. Furthermore, it is important to note that participants who responded to advertisements on POZ.com may not be representative of the broader US population living with HIV, introducing potential selection bias. Financial incentives for study participation may have influenced responses, potentially skewing the participant pool toward individuals with greater financial needs.

Another significant limitation is the reliance on self-reported adherence data in the control group, which is prone to bias. Self-reported measures, particularly in the context of health behaviors, can be subject to overreporting due to recall bias or social desirability, where participants may provide responses that they believe are more acceptable to researchers [69,70]. Although the SRSI scale demonstrated high internal consistency, discrepancies between self-reported adherence and CleverCap data suggest that participants’ actual behaviors differed. Some adjusted their adherence schedules based on personal beliefs, indicating that self-reported data may not fully reflect true adherence. In addition, the reliance on self-reported viral load data as an inclusion criterion may have introduced some inaccuracies, as nearly half of the sample reported being virally unsuppressed (threshold of ≥200 copies/ml) in the past 12 months despite being clinically undetectable at baseline. This difference suggests that participants may have misremembered or misreported their viral load history, highlighting a potential limitation in self-reported viral suppression measures.

Discrepancies between the planned and delivered intervention fidelity were observed, with several factors impeding full implementation. While the intervention was designed to provide consistent support through regular CHW sessions and timely CleverCap notifications, technical challenges, such as unreliable local telecommunication networks, affected the delivery of mHealth components, leading to missed or delayed notifications. In addition, participant engagement waned over time, with some individuals disengaging from CHW sessions and underusing the CleverCap device, a challenge commonly reported in mHealth interventions [71,72]. The older demographic of the study population introduced additional barriers, as many participants had difficulty programming medication alerts without CHW assistance, leading to missed doses. This aligns with previous research indicating that older adults frequently encounter usability issues with electronic adherence tools, which can negatively impact adherence [71]. These findings highlight the importance of tailoring adherence interventions to address technological literacy and providing structured training for older adults.

Finally, the lack of a universal data verification process for the DBS results posed a limitation. While DBS samples were collected and processed according to established protocols, most participants’ (25/30, 83%) DBS results were not cross verified with other sources. Only a small subset of participants (5/30, 17%) who had requested a screening visit and subsequently provided electronic health record viral load data had their DBS results cross-checked. This reliance on unverified DBS data for most participants may introduce potential inaccuracies. Furthermore, the moderate sensitivity (80%-95%) and specificity (85%-90%) of DBS for viral load thresholds, such as 200 to 1000 copies/ml, could lead to misclassification, with some participants incorrectly identified as having high or low viral loads [73-75]. Future studies should incorporate systematic verification processes, such as routine cross-checking with laboratory results or electronic health records, to enhance the reliability of biomarker data.

### Conclusions

The findings from this pilot study support the feasibility and acceptability of a remotely delivered mHealth intervention integrated with CHW support for people with HIV. Participants reported high levels of usability and satisfaction with both the WiseApp and CleverCap, highlighting the intervention’s potential to promote medication adherence and engagement in care. Despite modest, nonsignificant improvements in self-reported adherence and self-efficacy, the flexibility of the intervention and its ability to address logistical challenges make it a promising tool for broader implementation. These results suggest that with further optimization, this intervention could be a valuable resource in enhancing health outcomes for people with HIV across diverse settings.

## Appendix

Multimedia Appendix 1 Key themes, definitions, and illustrative quotes from control and intervention group participants, organized according to the Mobile Health Technology Acceptance Model.

Multimedia Appendix 2 Retention of participants at follow-up by baseline viral load status, assessing whether the loss to follow-up was differentially associated with baseline viral load in the intervention and control groups.

Multimedia Appendix 3 Methods of viral load and CD4 data collection at baseline and follow-up, including self-reported and laboratory measures.

Multimedia Appendix 4 CONSORT eHEALTH checklist (V 1.6.1).

## Abbreviations

aOR: adjusted odds ratio
ART: antiretroviral therapy
CHAMPS: Community Health Worker and mHealth to Improve Viral Suppression
CHW: community health worker
DBS: dried blood spot
eROI: electronic release of information
Health-ITUES: Health Information Technology Usability Evaluation Scale
HIPAA: Health Insurance Portability and Accountability Act
HIV-ASES: HIV Treatment Adherence Self-Efficacy Scale
mHealth: mobile health
MHTAM: Mobile Health Technology Acceptance Model
PSSUQ: Poststudy System Usability Questionnaire
REDCap: Research Electronic Data Capture
SRSI: self-reported scale item

## Data Availability

The datasets generated or analyzed during this study are not publicly available due to participant confidentiality and institutional data protection policies, but are available from the corresponding author on reasonable request.
