# Supplementary material for: A Remote Intervention Based on mHealth and Community Health Workers for Antiretroviral Therapy Adherence in People With HIV: Pilot Randomized Controlled Trial
Source: JMIR Form Res. 2025 Apr 2;9:e67997. doi: 10.2196/67997 (PMC12004026; doi:10.2196/67997)
Supplement: Multimedia Appendix 1 [file formative_v9i1e67997_app1.pdf]

SUPPLEMENTARY MATERIAL

Supplementary Table 1: Key themes, definitions, and illustrative quotes from control and intervention group participants, organized according to the MHTAM

Thematic Code	Illustrative Quote(s)
**(1.0) Perceived Usefulness:** The degree to which a person believes that using the CHAMPS intervention or participating in the study enhances their medication adherence and health outcomes	
(1.1.1) CHAMPS Intervention - Barrier	*I have to say for myself [the CHW sessions] weren't very helpful, simply because that wasn't available, and that's a bit unfortunate… again, as a result of my routine and of my schedule, they weren't very helpful for me… So, the idea that I felt badly about not being able to take the calls, or stay on track with the calls, was my experience. (CHP21)*
(1.1.2) CHAMPS Intervention - Facilitator	*I think [the CleverCap is] something that many people would like to utilize, including myself, for many different reasons. My reason being, as I stated before, me sometimes forgetting if I took it or not because my memory sometimes fails me. And I know a lot of other people try to stick with a regimen. And I'm not sure of their reasons for not being able to stick with their regimen. But the CleverCap seems like something that would help them stick to their regimen, and also improve their overall health more because it has helped me manage my other medications more. And it also kept me focused on my health even more than I am focused on. And so I believe it would serve as an intervention for prevention of health decline because the daily presence of it is a more conscious reminder of overall health, not just daily doses. (CHP35)*
(1.2.2) Study Procedures - Facilitator	*And so, when I applied, I was really excited. I thought I was maybe going to get the app and the pill box. But, I was put in the branch that doesn't, which is fine, but it has still been really helpful, because in the last six months, I have been able to achieve viral load that was undetectable three times. So, I really feel that you guys have helped me focus on my goal of trying to get undetectable. I'm really glad you guys are on my part to achieve, and you guys were able to help me. (CHP22)*
**(2.0) Perceived Ease of Use:** The degree to which a person believes that using the CHAMPS intervention or participating in the study is free of effort.	
(2.1.1) CHAMPS Intervention - Barrier	*Honestly, [the CleverCap] was somewhat too large. I wish they had it set up, which I know is…well, they have, modern technology and people creating new things and ideas. In terms of like, if it was in a case…or if it had pockets, for example. Like I said, if I'm taking more than one medication, you would want to have different pockets to put them in. You understand?... It would make it more convenient; you know? (CHP13)*

(2.1.2) CHAMPS Intervention - Facilitator	*And I found it also very helpful having the reminders… It was just one less thing for me to focus on as far as trying to remember if I took my medications or not. Because sometimes when I'm very stressed out with a lot on my mind, I sometimes forget if I took my medicine or not. But using the CleverCap, it alleviated that wonder if I took it or not. (CHP35)*
(2.2.1) Study Procedures - Barrier	*The blood doesn't flow. I don't know if it's because I'm older or what. I didn't get a lot of blood out of me. I had to do it three times, you know. But I don't know how else you could do it at home and it could work. So, I guess this is what has to be done. I don't know another way. (CHP30)*
(2.2.2) Study Procedures - Facilitator	*Well, doing the test, when you open the test kit, I think you have Band-Aids and everything in it. It explained to me what exactly to do – wash my hands. Everything was thorough. It told me what finger to prick and everything. It didn't hurt, but I thought it was going to hurt, because I had never done it before. After that, the directions, they helped, yes. It went smoothly. (CHP23)*
**(3.0) Perceived Ubiquity:** The degree to which a person believes that the CHAMPS intervention can be used anytime and anywhere, or study participation can occur from anywhere.	
(3.1.1) CHAMPS Intervention - Barrier	*Especially for a person who takes medication that involves something so personal that you don't really want other people to know about… [the CleverCap] just makes it more noticeable. I had a friend who noticed it lighting up in my purse one day…what's that? It becomes uncomfortable. I think some of that should be looked at. (CHP17)*
(3.1.2) CHAMPS Intervention - Facilitator	*When I went to D.C… I forgot the Clever Cap. And it was in the house going off. Got my texts. But it was in a different state... But I had my medicine. But you know I was just getting the notifications like, oh, it was time to take your medicine. Didn't bring the cap. (CHP32)*
(3.2.1) Study Procedures - Barrier	*Because, I don't always have transportation, so it's been harder for me to get the package sent back in like a timely manner. I know you guys have tried setting up a pick-up with FedEx, but they never came to pick up the package. So, that's like been the only barrier I've had in participating in the program and getting the package to a FedEx location. (CHP27)*
(3.2.2) Study Procedures - Facilitator	*I appreciate it by Zoom. Because it lets you be where, you know where you consider your safe space. To necessarily navigate the appointment and first of all, it's just what's in now, at this point. You know with it being like 2024. And you know contact-free type of situations. And I think, like you know, you can be in another part of the world, I can be here, or you know different time zones. And we can still come together and connect for a common cause and things. (CHP31)*

**(4.0) Self-efficacy:** The belief in one's own ability to successfully use the CHAMPS intervention and adhere to medication, or to successfully complete the study procedures.	
(4.1.1) CHAMPS Intervention - Barrier	*I'll be honest with you. I never even put the medication in [the CleverCap]. I kept it in its original container. I just had it at my disposal so I can just, when it went off, when it notified me, I was able to unscrew it. You know and take my medication and then put the top back on. (CHP13)*
(4.1.2) CHAMPS Intervention - Facilitator	*It would send me like the percentage, like 84% or 91% and I'm like, ok, well I'm doing good. But then, it was basically, I'm trying to compete with the app. So, I can get 100%. So, I'm thinking, you know you just kind of do better and be better with the CleverCap. (CHP32)*
(4.2.1) Study Procedures - Barrier	*You sent me a kit. I didn't use it because I didn't really pay… I'm good at following instructions sometimes. But I didn't know whether it was a needlestick. I didn't take time out to read it thoroughly. (CHP13)*
(4.2.2) Study Procedures - Facilitator	*I am generally interested in anything involving HIV because as I mentioned previously, I am a long-term survivor. I was diagnosed in June of '95. So, this is one way I give back. I am not here solely because of what I did. Basically, participation in HIV-related stuff is how I give back. I am not alive because of my own efforts. A lot of people helped me out over the years, so I owe it, and I owe it to all those who did pass away; so those who no longer have a voice. (CHP16)*
**(5.0) Technology Literacy:** The level of familiarity and comfort with using technology.	
(5.1.1) CHAMPS Intervention - Barrier	*Older people, we're not liking apps. Especially people who don't have a lot of knowledge with technical devices, electronic devices. (CHP38)*
(5.1.2) CHAMPS Intervention - Facilitator	*It was good for me. And I had CleverCap before, remember I told you. I wasn't persistent on taking it like I am now... (CHP04)*
(5.2.2) Study Procedures - Facilitator	*Yeah. But it's crazy because remember I told you I was in the CHAMPS study before, but it wasn't at home. You understand? I used to have to come in. I still had to do the questionnaire over the laptop. You know what I mean? And so, I already knew what I had to do anyway. (CHP04)*
**(6.0) Subjective Norms:** The influence of others' opinions, behaviors, and HIV stigma on the acceptance and use of the CHAMPS intervention or participating in the study.	
(6.1.1) CHAMPS Intervention - Barrier	*I didn't feel comfortable with taking [the CleverCap] outside my home, mainly because I know it's important at times to have medications in their prescribed bottles. And so, if I was to come across a situation with an officer. I haven't been at an airport. But I need to have it in the prescribed bottle. I didn't want to have to go into any explanation with anyone. (CHP35)*

(6.1.2) CHAMPS Intervention - Facilitator	*[The study] really was awesome. I mean the perk was having you call me once a week. I know I can't have that now. Because that's like, oh my god, that would be great. Having your own personal somebody to motivate you and check up on you and make sure you're doing good and adhering to your medicine. (CHP39)*
(6.2.1) Study Procedures - Barrier	*You might want to ask a little bit more if a person has internalized stigma. A lot of what goes on outside… but then, some people can have a ton of support from outside and still feel that they've done something wrong internal and still feel not right internally. (CHP16)*
(6.2.2) Study Procedures - Facilitator	*I wasn't really sure what [the blood collection] was going to entail, so I was nervous, which is why I made my boyfriend help me and come with me. (CHP17)*
