# Supplementary material for: A Remote Intervention Based on mHealth and Community Health Workers for Antiretroviral Therapy Adherence in People With HIV: Pilot Randomized Controlled Trial
Source: JMIR Form Res. 2025 Apr 2;9:e67997. doi: 10.2196/67997 (PMC12004026; doi:10.2196/67997)
Supplement: Multimedia Appendix 2 [file formative_v9i1e67997_app2.pdf]

Supplementary Table 2: Retention of participants at follow-up by baseline viral load status, assessing whether loss to follow-up was differentially associated with baseline viral load in the intervention and control groups

	Control			Intervention		
Baseline Viral Load Status	Follow-up status			Follow-up status		
	Completed	Lost	Total	Completed	Lost	Total
**Not detectable**	14	3	17	7	4	11
**Detectable**	3	0	3	5	4	9
**Total**	17	3	20	12	8	20
