# Supplementary material for: A Remote Intervention Based on mHealth and Community Health Workers for Antiretroviral Therapy Adherence in People With HIV: Pilot Randomized Controlled Trial
Source: JMIR Form Res. 2025 Apr 2;9:e67997. doi: 10.2196/67997 (PMC12004026; doi:10.2196/67997)
Supplement: Multimedia Appendix 3 [file formative_v9i1e67997_app3.pdf]

Supplementary Table 3: Methods of viral load and CD4 data collection at baseline and follow-up, including self-reported and laboratory measures

Method for Obtaining Viral Load and CD4	Baseline n(%)	Follow-up n(%)
**Participant-Uploaded Result**	25 (62.5)	14 (46.67)
**Dried Blood Spot**	14 (35)	16 (53.33)
**eROI/HIPAA**	1 (2.5)	0 (0)
**Total**	40	30
