# Supplementary material for: A Remote Intervention Based on mHealth and Community Health Workers for Antiretroviral Therapy Adherence in People With HIV: Pilot Randomized Controlled Trial
Source: JMIR Form Res. 2025 Apr 2;9:e67997. doi: 10.2196/67997 (PMC12004026; doi:10.2196/67997)
Supplement: Multimedia Appendix 4 [file formative_v9i1e67997_app4.pdf]

CONSORT-EHEALTH (V 1.6.1) - Submission/Publication Form

The CONSORT-EHEALTH checklist is intended for authors of randomized trials evaluating web-based and Internet-based applications/interventions, including mobile interventions, electronic games (incl multiplayer games), social media, certain telehealth applications, and other interactive and/or networked electronic applications. Some of the items (e.g. all subitems under item 5 - description of the intervention) may also be applicable for other study designs.

The goal of the CONSORT EHEALTH checklist and guideline is to be
a) a guide for reporting for authors of RCTs,
b) to form a basis for appraisal of an ehealth trial (in terms of validity)

CONSORT-EHEALTH items/subitems are MANDATORY reporting items for studies published in the Journal of Medical Internet Research and other journals / scientific societies endorsing the checklist.

Items numbered 1., 2., 3., 4a., 4b etc are original CONSORT or CONSORT-NPT (non-pharmacologic treatment) items.
Items with Roman numerals (i., ii, iii, iv etc.) are CONSORT-EHEALTH extensions/clarifications.

As the CONSORT-EHEALTH checklist is still considered in a formative stage, we would ask that you also RATE ON A SCALE OF 1-5 how important/useful you feel each item is FOR THE PURPOSE OF THE CHECKLIST and reporting guideline (optional).

Mandatory reporting items are marked with a red *.
In the textboxes, either copy & paste the relevant sections from your manuscript into this form - please include any quotes from your manuscript in QUOTATION MARKS,
or answer directly by providing additional information not in the manuscript, or elaborating on why the item was not relevant for this study.

YOUR ANSWERS WILL BE PUBLISHED AS A SUPPLEMENTARY FILE TO YOUR PUBLICATION IN JMIR AND ARE CONSIDERED PART OF YOUR PUBLICATION (IF ACCEPTED).
Please fill in these questions diligently. Information will not be copyedited, so please use proper spelling and grammar, use correct capitalization, and avoid abbreviations.

DO NOT FORGET TO SAVE AS PDF _AND_ CLICK THE SUBMIT BUTTON SO YOUR ANSWERS ARE IN OUR DATABASE !!!

Your response is too large. Try shortening some answers.

Eysenbach G, CONSORT-EHEALTH Group

CONSORT-EHEALTH: Improving and Standardizing Evaluation Reports of Web-based and Mobile Health Interventions
J Med Internet Res 2011;13(4):e126
URL: http://www.jmir.org/2011/4/e126/
doi: 10.2196/jmir.1923
PMID: 22209829

sshourya9@gmail.com Switch account

Not shared

Draft saved

* Indicates required question

Your name *

First Last

Shivesh Shourya

Primary Affiliation (short), City, Country *

University of Toronto, Toronto, Canada

Yale University, New Haven, United States of A[illegible]

Your e-mail address *

abc@gmail.com

shivesh.shourya@yale.edu

Title of your manuscript *

Provide the (draft) title of your manuscript.

A Remote mHealth and CHW-Based Intervention (CHAMPS) for ART Adherence in People

Your response is too large. Try shortening some answers.

Name of your App/Software/Intervention *

If there is a short and a long/alternate name, write the short name first and add the long name in brackets.

WiseApp (or CleverCap)

Evaluated Version (if any)

e.g. "V1", "Release 2017-03-01", "Version 2.0.27913"

Your answer

Language(s) *

What language is the intervention/app in? If multiple languages are available, separate by comma (e.g. "English, French")

English

URL of your Intervention Website or App

e.g. a direct link to the mobile app on app in appstore (itunes, Google Play), or URL of the website. If the intervention is a DVD or hardware, you can also link to an Amazon page.

https://play.google.com/store/apps/details?id=com.clevercap.enterprise.mobileappui&hl=en

URL of an image/screenshot (optional)

Your answer

Your response is too large. Try shortening some answers.

Accessibility *

Can an enduser access the intervention presently?

- (selected) access is free and open
- access only for special usergroups, not open
- access is open to everyone, but requires payment/subscription/in-app purchases
- app/intervention no longer accessible
- Other:

Primary Medical Indication/Disease/Condition *

e.g. "Stress", "Diabetes", or define the target group in brackets after the condition, e.g. "Autism (Parents of children with)", "Alzheimers (Informal Caregivers of)"

Living with Human Immunodeficiency Virus (H

Primary Outcomes measured in trial *

comma-separated list of primary outcomes reported in the trial

Feasibility of the CHAMPS (Community Health

Secondary/other outcomes

Are there any other outcomes the intervention is expected to affect?

ART adherence (SRSI), Usability and Acceptability of the intervention (Health-ITUES and PSSUQ), feedback via interviews

Your response is too large. Try shortening some answers.

Recommended "Dose" *

What do the instructions for users say on how often the app should be used?

- (selected) Approximately Daily
- Approximately Weekly
- Approximately Monthly
- Approximately Yearly
- "as needed"
- Other:

Approx. Percentage of Users (starters) still using the app as recommended after 3 months *

- (selected) unknown / not evaluated
- 0-10%
- 11-20%
- 21-30%
- 31-40%
- 41-50%
- 51-60%
- 61-70%
- 71%-80%
- 81-90%
- 91-100%

Your response is too large. Try shortening some answers.

Overall, was the app/intervention effective? *

- ○ yes: all primary outcomes were significantly better in intervention group vs control
- ○ partly: SOME primary outcomes were significantly better in intervention group vs control
- ◉ no statistically significant difference between control and intervention
- ○ potentially harmful: control was significantly better than intervention in one or more outcomes
- ○ inconclusive: more research is needed
- ○ Other:

Article Preparation Status/Stage *

At which stage in your article preparation are you currently (at the time you fill in this form)

- ○ not submitted yet - in early draft status
- ○ not submitted yet - in late draft status, just before submission
- ○ submitted to a journal but not reviewed yet
- ○ submitted to a journal and after receiving initial reviewer comments
- ○ submitted to a journal and accepted, but not published yet
- ○ published
- ◉ Other: submitted to a journal and have recieved second round of reviewer co

Your response is too large. Try shortening some answers.

Journal *

If you already know where you will submit this paper (or if it is already submitted), please provide the journal name (if it is not JMIR, provide the journal name under "other")

- ○ not submitted yet / unclear where I will submit this
- ○ Journal of Medical Internet Research (JMIR)
- ○ JMIR mHealth and UHealth
- ○ JMIR Serious Games
- ○ JMIR Mental Health
- ○ JMIR Public Health
- ◉ JMIR Formative Research
- ○ Other JMIR sister journal
- ○ Other:

Is this a full powered effectiveness trial or a pilot/feasibility trial? *

- ◉ Pilot/feasibility
- ○ Fully powered

Manuscript tracking number *

If this is a JMIR submission, please provide the manuscript tracking number under "other" (The ms tracking number can be found in the submission acknowledgement email, or when you login as author in JMIR. If the paper is already published in JMIR, then the ms tracking number is the four-digit number at the end of the DOI, to be found at the bottom of each published article in JMIR)

- ○ no ms number (yet) / not (yet) submitted to / published in JMIR

Your response is too large. Try shortening some answers.

TITLE AND ABSTRACT

1a) TITLE: Identification as a randomized trial in the title

1a) Does your paper address CONSORT item 1a? *

I.e does the title contain the phrase "Randomized Controlled Trial"? (if not, explain the reason under "other")

- (selected) yes
- Other:

1a-i) Identify the mode of delivery in the title

Identify the mode of delivery. Preferably use “web-based” and/or “mobile” and/or “electronic game” in the title. Avoid ambiguous terms like “online”, “virtual”, “interactive”. Use “Internet-based” only if Intervention includes non-web-based Internet components (e.g. email), use “computer-based” or “electronic” only if offline products are used. Use “virtual” only in the context of “virtual reality” (3-D worlds). Use “online” only in the context of “online support groups”. Complement or substitute product names with broader terms for the class of products (such as “mobile” or “smart phone” instead of “iphone”), especially if the application runs on different platforms.

	1	2	3	4	5	
subitem not at all important	○	○	○	○	●	essential

Clear selection

Your response is too large. Try shortening some answers.

Does your paper address subitem 1a-i? *

Copy and paste relevant sections from manuscript title (include quotes in quotation marks "like this" to indicate direct quotes from your manuscript), or elaborate on this item by providing additional information not in the ms, or briefly explain why the item is not applicable/relevant for your study

Manuscript Title: A Remote mHealth and CHW-Based Intervention (CHAMPS) for ART Adherence in People with HIV: Findings from a Pilot Randomized Controlled Trial

Mode of Delivery: "Remote"

1a-ii) Non-web-based components or important co-interventions in title

Mention non-web-based components or important co-interventions in title, if any (e.g., “with telephone support”).

	1	2	3	4	5	
subitem not at all important	○	○	◉	○	○	essential

Clear selection

Does your paper address subitem 1a-ii?

Copy and paste relevant sections from manuscript title (include quotes in quotation marks "like this" to indicate direct quotes from your manuscript), or elaborate on this item by providing additional information not in the ms, or briefly explain why the item is not applicable/relevant for your study

Manuscript Title: A Remote mHealth and CHW-Based Intervention (CHAMPS) for ART Adherence in People with HIV: Findings from a Pilot Randomized Controlled Trial

Important co-intervention: "CHW-based" (community health worker)

Your response is too large. Try shortening some answers.

1a-iii) Primary condition or target group in the title

Mention primary condition or target group in the title, if any (e.g., “for children with Type I Diabetes”) Example: A Web-based and Mobile Intervention with Telephone Support for Children with Type I Diabetes: Randomized Controlled Trial

	1	2	3	4	5	
subitem not at all important	○	○	○	○	◉	essential

Clear selection

Does your paper address subitem 1a-iii? *

Copy and paste relevant sections from manuscript title (include quotes in quotation marks "like this" to indicate direct quotes from your manuscript), or elaborate on this item by providing additional information not in the ms, or briefly explain why the item is not applicable/relevant for your study

Manuscript Title: A Remote mHealth and CHW-Based Intervention (CHAMPS) for ART Adherence in People with HIV: Findings from a Pilot Randomized Controlled Trial

Target group: "Peoplw with HIV"

1b) ABSTRACT: Structured summary of trial design, methods, results, and conclusions

NPT extension: Description of experimental treatment, comparator, care providers, centers, and blinding status.

Your response is too large. Try shortening some answers.

1b-i) Key features/functionalities/components of the intervention and comparator in the METHODS section of the ABSTRACT

Mention key features/functionalities/components of the intervention and comparator in the abstract. If possible, also mention theories and principles used for designing the site. Keep in mind the needs of systematic reviewers and indexers by including important synonyms. (Note: Only report in the abstract what the main paper is reporting. If this information is missing from the main body of text, consider adding it)

	1	2	3	4	5	
subitem not at all important	○	○	○	○	◉	essential

Clear selection

Does your paper address subitem 1b-i? *

Copy and paste relevant sections from the manuscript abstract (include quotes in quotation marks "like this" to indicate direct quotes from your manuscript), or elaborate on this item by providing additional information not in the ms, or briefly explain why the item is not applicable/relevant for your study

"Objective: This pilot study evaluated the feasibility, acceptability, and preliminary efficacy of remote delivery of the CHAMPS intervention, combining the WiseApp, CHW support, and the CleverCap smart pill bottle."

1b-ii) Level of human involvement in the METHODS section of the ABSTRACT

Clarify the level of human involvement in the abstract, e.g., use phrases like “fully automated” vs. “therapist/nurse/care provider/physician-assisted” (mention number and expertise of providers involved, if any). (Note: Only report in the abstract what the main paper is reporting. If this information is missing from the main body of text, consider adding it)

	1	2	3	4	5	
subitem not at all important	○	○	○	◉	○	essential

Your response is too large. Try shortening some answers.

Does your paper address subitem 1b-ii?

Copy and paste relevant sections from the manuscript abstract (include quotes in quotation marks "like this" to indicate direct quotes from your manuscript), or elaborate on this item by providing additional information not in the ms, or briefly explain why the item is not applicable/relevant for your study

"The intervention group participated in up to twelve sessions with CHWs and used the WiseApp, paired with a CleverCap smart pill bottle, to support ART adherence."

1b-iii) Open vs. closed, web-based (self-assessment) vs. face-to-face assessments in the METHODS section of the ABSTRACT

Mention how participants were recruited (online vs. offline), e.g., from an open access website or from a clinic or a closed online user group (closed usergroup trial), and clarify if this was a purely web-based trial, or there were face-to-face components (as part of the intervention or for assessment). Clearly say if outcomes were self-assessed through questionnaires (as common in web-based trials). Note: In traditional offline trials, an open trial (open-label trial) is a type of clinical trial in which both the researchers and participants know which treatment is being administered. To avoid confusion, use “blinded” or “unblinded” to indicated the level of blinding instead of “open”, as “open” in web-based trials usually refers to “open access” (i.e. participants can self-enrol). (Note: Only report in the abstract what the main paper is reporting. If this information is missing from the main body of text, consider adding it)

	1	2	3	4	5	
subitem not at all important	○	◉	○	○	○	essential

Clear selection

Your response is too large. Try shortening some answers.

Does your paper address subitem 1b-iii?

Copy and paste relevant sections from the manuscript abstract (include quotes in quotation marks "like this" to indicate direct quotes from your manuscript), or elaborate on this item by providing additional information not in the ms, or briefly explain why the item is not applicable/relevant for your study

Participants were recruited from a plethora of websites including POZ.com, Facebook, Craigslist; but they were also recrutied via study team efforts outreaching to various clinics to post advertisemenst.

1b-iv) RESULTS section in abstract must contain use data

Report number of participants enrolled/assessed in each group, the use/uptake of the intervention (e.g., attrition/adherence metrics, use over time, number of logins etc.), in addition to primary/secondary outcomes. (Note: Only report in the abstract what the main paper is reporting. If this information is missing from the main body of text, consider adding it)

	1	2	3	4	5	
subitem not at all important	○	○	○	○	●	essential

Clear selection

Your response is too large. Try shortening some answers.

Does your paper address subitem 1b-iv?

Copy and paste relevant sections from the manuscript abstract (include quotes in quotation marks "like this" to indicate direct quotes from your manuscript), or elaborate on this item by providing additional information not in the ms, or briefly explain why the item is not applicable/relevant for your study

"This mixed-methods pilot study involved 40 participants (n = 20 control, n = 20 intervention) "

"Remote delivery of the CHAMPS intervention is feasible, with high usability ratings for both the WiseApp and CleverCap (Health-ITUES overall score: mean = 4.35, SD ± 0.58; PSSUQ overall score: mean = 2.04, SD ± 1.03). There were non-significant improvements in self-reported adherence (SRSI: intervention group baseline mean = 4.90, follow-up mean = 5.25, p = 0.288) and self-efficacy (HIV-ASES overall score: intervention group baseline mean = 8.08, follow-up mean = 8.78, p = 0.072). The adjusted odds ratio (aOR) for achieving undetectable viral load in the intervention group compared to the control group was 3.01, indicating a medium effect size in favor of the intervention. Overall study retention was 75% (n = 30), with higher retention in the control group. Participants valued the flexibility of remote study procedures, particularly Zoom-based study visits and mailed blood sample kits. Qualitative feedback highlighted the intervention's acceptability and its ability to overcome logistical barriers."

1b-v) CONCLUSIONS/DISCUSSION in abstract for negative trials

Conclusions/Discussions in abstract for negative trials: Discuss the primary outcome - if the trial is negative (primary outcome not changed), and the intervention was not used, discuss whether negative results are attributable to lack of uptake and discuss reasons. (Note: Only report in the abstract what the main paper is reporting. If this information is missing from the main body of text, consider adding it)

	1	2	3	4	5	
subitem not at all important	○	○	○	◉	○	essential

Clear selection

Your response is too large. Try shortening some answers.

Does your paper address subitem 1b-v?

Copy and paste relevant sections from the manuscript abstract (include quotes in quotation marks "like this" to indicate direct quotes from your manuscript), or elaborate on this item by providing additional information not in the ms, or briefly explain why the item is not applicable/relevant for your study

"The remote CHAMPS pilot study demonstrated feasibility and acceptability of combining mHealth tools with CHW support to promote medication adherence among PWH. While further optimization is needed to enhance its impact, this intervention shows potential for improving health outcomes in diverse, underserved populations."

INTRODUCTION

2a) In INTRODUCTION: Scientific background and explanation of rationale

2a-i) Problem and the type of system/solution

Describe the problem and the type of system/solution that is object of the study: intended as stand-alone intervention vs. incorporated in broader health care program? Intended for a particular patient population? Goals of the intervention, e.g., being more cost-effective to other interventions, replace or complement other solutions? (Note: Details about the intervention are provided in “Methods” under 5)

	1	2	3	4	5	
subitem not at all important	○	○	○	○	◉	essential

Clear selection

Your response is too large. Try shortening some answers.

Does your paper address subitem 2a-i? *

Copy and paste relevant sections from the manuscript (include quotes in quotation marks "like this" to indicate direct quotes from your manuscript), or elaborate on this item by providing additional information not in the ms, or briefly explain why the item is not applicable/relevant for your study

"With the advent of antiretroviral therapy (ART) for HIV, people with HIV (PWH) can now achieve near-normal life expectancies[1-4]. At the end of 2021, over 50% of PWH in the United States (US) were aged ≥ 50 and older, largely a result of the high efficacy of ART[5]. However, rates of viral suppression among the 1.2 million PWH in the US continue to remain low with current estimates around 66% among PWH, despite wider availability of ART[5]. The suboptimal rates of viral suppression may be attributed to poor ART adherence [6] and come at a time of increasing healthcare fragmentation, leading to higher costs of care and lower health outcomes for many chronically ill patients such as PWH[7].
Ending the HIV Epidemic (EHE) plan, identifies viral suppression as a cornerstone to preventing and treating HIV infections[8-10]. HIV viral suppression is dependent on adherence to ART, and studies demonstrate that high rates of ART adherence (~95%) are attributed to higher rates of viral suppression (~78%)[11]. However, even moderate decreases in adherence to ART (from 95% to 80%) can lead to drastic reductions in viral suppression rates (from 78% to 20%), underscoring that ART adherence be maintained 95% or higher[11]. Poor adherence to ART can lead to poor outcomes for the patient, emergence of drug-resistant HIV strains[12], and increased rates of HIV transmission[13]. Suboptimal levels of ART adherence, along with low levels of engagement in the HIV care continuum, have also been attributed to the progression of HIV disease and premature deaths among PWH[14]. Therefore, there is an urgent need to develop and evaluate interventions that enhance adherence to ART.
Developing interventions to increase ART adherence must address prevalent barriers to optimal ART adherence by championing a comprehensive approach to understanding and addressing unmet needs of PWH. Previous studies have identified numerous barriers to sustained adherence to ART, engagement with medical care, and, consequently, viral suppression, including: HIV stigma or fear of HIV status disclosure[15-19], geographic barriers or limited time resources for receiving/accessing HIV care[16, 17, 19], negative experiences within medical institutions or HIV care centers[15, 16, 19], and forgetfulness[18, 19]. Similar studies have outlined strong networks of social support, HIV case managers, telemedicine appointments, and reminder tools as facilitators of ART adherence and medical care engagement[16-19]. "

Your response is too large. Try shortening some answers.

2a-ii) Scientific background, rationale: What is known about the (type of) system

Scientific background, rationale: What is known about the (type of) system that is the object of the study (be sure to discuss the use of similar systems for other conditions/diagnoses, if appropiate), motivation for the study, i.e. what are the reasons for and what is the context for this specific study, from which stakeholder viewpoint is the study performed, potential impact of findings [2]. Briefly justify the choice of the comparator.

	1	2	3	4	5	
subitem not at all important	○	○	○	○	◉	essential

Clear selection

Your response is too large. Try shortening some answers.

Does your paper address subitem 2a-ii? *

Copy and paste relevant sections from the manuscript (include quotes in quotation marks "like this" to indicate direct quotes from your manuscript), or elaborate on this item by providing additional information not in the ms, or briefly explain why the item is not applicable/relevant for your study

"Recently, there has been an increased focus on the utility of community health workers (CHW), who can take on roles of outreach workers, patient navigators, health advisors, or peer leaders in helping access and manage HIV in primary care settings[20]. While the profile, skill level, and job scope of CHWs can vary widely based on context, they often serve as patient navigators and peer educators in the US, focusing on HIV care retention, ART adherence, and viral suppression support, particularly in under resourced communities[21-23]. Several studies have found that integrating CHWs was associated with improved care retention, ART adherence, and viral suppression outcomes[20, 24, 25], nonetheless, other studies have found no significant difference in viral suppression outcomes between PWH assigned CHWs and PWH without CHW interventionists[26, 27]. While some studies failed to show statistical significance in viral suppression outcomes between intervention and control groups, they emphasized the utility of a combined intervention including CHW components and other interventions to address barriers to ART adherence[26, 27]. Mobile health (mHealth) interventions offer themselves as novel interventions in HIV care settings given their ubiquity, ability to overcome geographic barriers to care, and low associated costs[28]. However, previous studies have provided mixed results, with some finding increased viral suppression and retention in care rates among mHealth intervention participants [29, 30] and others finding no statistical significant difference in viral suppression and retention rates between intervention and control participants[31]. Previous study limitations include poor integration of mHealth technologies within HIV primary care settings, and researchers have advocated for a combination approach with patient navigation services to help prioritize human communications and achieve desired outcomes related to viral suppression[31]. Thus, interventions that combine the use of CHWs and mHealth technologies hold promise for increasing rates of ART adherence and viral suppression within the US[24, 32]. "

2b) In INTRODUCTION: Specific objectives or hypotheses

Your response is too large. Try shortening some answers.

Does your paper address CONSORT subitem 2b? *

Copy and paste relevant sections from the manuscript (include quotes in quotation marks "like this" to indicate direct quotes from your manuscript), or elaborate on this item by providing additional information not in the ms, or briefly explain why the item is not applicable/relevant for your study

"In this pilot study titled, Community Health Worker And MHealth to ImProve Viral Suppression (CHAMPS remote), conducted by our team at the Columbia University School of Nursing, we tested the feasibility of a remotely delivered CHW intervention through an mHealth app, WiseApp. Participants were recruited from across the United States. Participants were provided with a smart pill bottle, CleverCap, that interfaced with the WiseApp for personalized medication reminders[33, 34]. We also gathered feedback on acceptability of remotely conducted study procedures."

METHODS

3a) Description of trial design (such as parallel, factorial) including allocation ratio

Does your paper address CONSORT subitem 3a? *

Copy and paste relevant sections from the manuscript (include quotes in quotation marks "like this" to indicate direct quotes from your manuscript), or elaborate on this item by providing additional information not in the ms, or briefly explain why the item is not applicable/relevant for your study

Our trial used a 1:1 allocation ratio as can be seen with the sample sizes per group, it was a two-arm (or parallel) RCT

"The pilot study was a two-arm randomized control trial (RCT) among 40 PWH who were followed over a three-month period. Participants were randomly assigned to CHAMPS (intervention) (n = 20) or a standard-of-care (control) (n = 20) arm."

3b) Important changes to methods after trial commencement (such as eligibility criteria) with reasons

Your response is too large. Try shortening some answers.

Does your paper address CONSORT subitem 3b? *

Copy and paste relevant sections from the manuscript (include quotes in quotation marks "like this" to indicate direct quotes from your manuscript), or elaborate on this item by providing additional information not in the ms, or briefly explain why the item is not applicable/relevant for your study

N/A no changes to methods were made after trial commencement

3b-i) Bug fixes, Downtimes, Content Changes

Bug fixes, Downtimes, Content Changes: ehealth systems are often dynamic systems. A description of changes to methods therefore also includes important changes made on the intervention or comparator during the trial (e.g., major bug fixes or changes in the functionality or content) (5-iii) and other “unexpected events” that may have influenced study design such as staff changes, system failures/downtimes, etc. [2].

	1	2	3	4	5	
subitem not at all important	◉	○	○	○	○	essential

Clear selection

Does your paper address subitem 3b-i?

Copy and paste relevant sections from the manuscript (include quotes in quotation marks "like this" to indicate direct quotes from your manuscript), or elaborate on this item by providing additional information not in the ms, or briefly explain why the item is not applicable/relevant for your study

N/A no changes to methods were made after trial commencement

4a) Eligibility criteria for participants

Your response is too large. Try shortening some answers.

Does your paper address CONSORT subitem 4a? *

Copy and paste relevant sections from the manuscript (include quotes in quotation marks "like this" to indicate direct quotes from your manuscript), or elaborate on this item by providing additional information not in the ms, or briefly explain why the item is not applicable/relevant for your study

"Participants were eligible to participate if they 1) were able to speak, read, and write in English; 2) were aged ≥18 years; 3) were willing to provide a valid form of identification for verification; 4) were willing to participate in any assigned arm of the intervention; 5) were diagnosed with HIV ≥6 months ago; 6) had an HIV-1 RNA level >50 copies/mL or reported being virally unsuppressed in the past 12 months (≥200 copies/mL); 7) were owners of a smartphone; 8) were capable and willing to provide informed consent for study participation and consent for access to medical records; and 9) lived in the US... Participants were not eligible if they meet any exclusion criteria, including: 1) residing in a nursing home, prison, and/or receiving in-patient psychiatric care at time of enrollment; 2) terminal illness with life expectancy <3 months; 3) planning to move out of the US in the next three months; and/or 4) participating in a study that targets viral suppression for PWH. "

4a-i) Computer / Internet literacy

Computer / Internet literacy is often an implicit “de facto” eligibility criterion - this should be explicitly clarified.

	1	2	3	4	5	
subitem not at all important	○	○	◉	○	○	essential

Clear selection

Does your paper address subitem 4a-i?

Copy and paste relevant sections from the manuscript (include quotes in quotation marks "like this" to indicate direct quotes from your manuscript), or elaborate on this item by providing additional information not in the ms, or briefly explain why the item is not applicable/relevant for your study

"7) were owners of a smartphone" implies some basic level of computer/mobile literacy

Your response is too large. Try shortening some answers.

4a-ii) Open vs. closed, web-based vs. face-to-face assessments:

Open vs. closed, web-based vs. face-to-face assessments: Mention how participants were recruited (online vs. offline), e.g., from an open access website or from a clinic, and clarify if this was a purely web-based trial, or there were face-to-face components (as part of the intervention or for assessment), i.e., to what degree got the study team to know the participant. In online-only trials, clarify if participants were quasi-anonymous and whether having multiple identities was possible or whether technical or logistical measures (e.g., cookies, email confirmation, phone calls) were used to detect/prevent these.

	1	2	3	4	5	
subitem not at all important	○	○	○	◉	○	essential

Clear selection

Does your paper address subitem 4a-ii? *

Copy and paste relevant sections from the manuscript (include quotes in quotation marks "like this" to indicate direct quotes from your manuscript), or elaborate on this item by providing additional information not in the ms, or briefly explain why the item is not applicable/relevant for your study

This was online recruitment. Please see the following quote from the ms:

"Participants were recruited via online advertisements posted on POZ.com, Craigslist, Facebook, and Instagram. Additionally, the study team emailed community-based organizations serving PWH across the country with requests to hang study flyers. To determine preliminary eligibility, participants either filled out a screener online or were screened by phone. Participants who filled out online screeners were followed up with phone calls and were given detailed instructions regarding the study prior to scheduling study visits."

Your response is too large. Try shortening some answers.

4a-iii) Information giving during recruitment

Information given during recruitment. Specify how participants were briefed for recruitment and in the informed consent procedures (e.g., publish the informed consent documentation as appendix, see also item X26), as this information may have an effect on user self-selection, user expectation and may also bias results.

	1	2	3	4	5	
subitem not at all important	○	○	○	◉	○	essential

Clear selection

Does your paper address subitem 4a-iii?

Copy and paste relevant sections from the manuscript (include quotes in quotation marks "like this" to indicate direct quotes from your manuscript), or elaborate on this item by providing additional information not in the ms, or briefly explain why the item is not applicable/relevant for your study

"Participants who filled out online screeners were followed up with phone calls and were given detailed instructions regarding the study prior to scheduling study visits."

4b) Settings and locations where the data were collected

Your response is too large. Try shortening some answers.

Does your paper address CONSORT subitem 4b? *

Copy and paste relevant sections from the manuscript (include quotes in quotation marks "like this" to indicate direct quotes from your manuscript), or elaborate on this item by providing additional information not in the ms, or briefly explain why the item is not applicable/relevant for your study

Your response is too large. Try shortening some answers.

"The study consisted of two required visits namely a baseline and a 3-month follow up visit. The study also consisted of an optional screening visit for participants who could not provide confirmation of viral load for eligibility. All study visits were conducted using secure HIPAA compliant Zoom technology. At the start of each study visit, participants were required to show a valid form of identification for verification and to avert fraud.

Screening visit

Screening visits lasted approximately one hour. Participants provided informed consent electronically through the Research Electronic Data Capture (REDCap) system, a secure platform for data collection[40, 41]. Before consenting, participants had the opportunity to ask study staff any questions about the process. Prior to the screening visit, participants were sent a dried blood spot (DBS) collection kit, along with detailed instructions. Trained study staff guided participants through the DBS collection process, which included the use of a BD Microtainer® Contact-Activated Lancet Blade (1.5mm X 2.0mm, Blue High Flow) to collect the sample. Care instructions for the puncture site were provided to ensure proper aftercare. Participants were instructed on how to package and ship their samples back to the lab at The Miriam Hospital in Rhode Island for processing. Upon receipt of lab results, study staff followed up with participants to inform them of their eligibility for the study. Compensation was not provided for screening visits.

Baseline visit

Baseline visits lasted two hours. Participants signed e-consent through REDCap, with the opportunity to ask study staff any questions prior to participating in the baseline visit. Upon enrollment, participants were randomized to either intervention or control arms through a randomization module in REDCap. Intervention participants were informed that they will be sent a CleverCap bottle and will have up to twelve study sessions over the duration of the study with an assigned CHW. Participants were required to complete a comprehensive survey via Qualtrics. The survey gathered information on demographics, a single item ART adherence self-reported scale item (SRSI)[42], and self-efficacy as a mediator of HIV treatment adherence (HIV-ASES)[43]. Baseline viral load and CD4 counts were recorded from information obtained via eROI, participant self-upload of lab results, or screening visit. Participants were compensated $40 in the form of an Amazon gift code for their time.

3-month follow-up visit

Follow-up visits lasted one hour. Participants completed a similar survey as the one administered at baseline with the addition of measures for usability displayed to intervention participants including the Health Information Technology Usability Evaluation Scale (Health-ITUES)[44] and the post-study system usability questionnaire (PSSUQ)[45]. Participants also provided viral load and CD4 counts obtained via eROI, participant self-upload of lab results, or through DBS collection. Real time adherence data from the CleverCap pill bottles was downloaded from the CleverCap website. At the end of the follow up visit, all participants were given the option to participate in an optional qualitative interview about their experience in the study and the perceived utility of a remotely delivered CHAMPS intervention. Follow-up interviews were conducted exclusively using secure HIPAA compliant Zoom technology and were audio-recorded for transcription. Consent for follow-up interviews was included in the study consent offered to participants at the baseline visit. Participants were compensated $50 for their time in the follow-up visit and an additional $35 if they opted to participate in the interview. Compensation was provided in the form of

Your response is too large. Try shortening some answers.

4b-i) Report if outcomes were (self-)assessed through online questionnaires

Clearly report if outcomes were (self-)assessed through online questionnaires (as common in web-based trials) or otherwise.

	1	2	3	4	5	
subitem not at all important	○	○	●	○	○	essential

Clear selection

Your response is too large. Try shortening some answers.

Does your paper address subitem 4b-i? *

Copy and paste relevant sections from the manuscript (include quotes in quotation marks "like this" to indicate direct quotes from your manuscript), or elaborate on this item by providing additional information not in the ms, or briefly explain why the item is not applicable/relevant for your study

Your response is too large. Try shortening some answers.

Some outcomes were self-assessed (Qualtrics questionnaire), whereas others such as HIV viral load could be self-reported or collected via a dried blood kit (self-collect but processed by a lab)

"The study consisted of two required visits namely a baseline and a 3-month follow up visit. The study also consisted of an optional screening visit for participants who could not provide confirmation of viral load for eligibility. All study visits were conducted using secure HIPAA compliant Zoom technology. At the start of each study visit, participants were required to show a valid form of identification for verification and to avert fraud.
Screening visit
Screening visits lasted approximately one hour. Participants provided informed consent electronically through the Research Electronic Data Capture (REDCap) system, a secure platform for data collection[40, 41]. Before consenting, participants had the opportunity to ask study staff any questions about the process. Prior to the screening visit, participants were sent a dried blood spot (DBS) collection kit, along with detailed instructions. Trained study staff guided participants through the DBS collection process, which included the use of a BD Microtainer® Contact-Activated Lancet Blade (1.5mm X 2.0mm, Blue High Flow) to collect the sample. Care instructions for the puncture site were provided to ensure proper aftercare. Participants were instructed on how to package and ship their samples back to the lab at The Miriam Hospital in Rhode Island for processing. Upon receipt of lab results, study staff followed up with participants to inform them of their eligibility for the study. Compensation was not provided for screening visits.
Baseline visit
Baseline visits lasted two hours. Participants signed e-consent through REDCap, with the opportunity to ask study staff any questions prior to participating in the baseline visit. Upon enrollment, participants were randomized to either intervention or control arms through a randomization module in REDCap. Intervention participants were informed that they will be sent a CleverCap bottle and will have up to twelve study sessions over the duration of the study with an assigned CHW. Participants were required to complete a comprehensive survey via Qualtrics. The survey gathered information on demographics, a single item ART adherence self-reported scale item (SRSI)[42], and self-efficacy as a mediator of HIV treatment adherence (HIV-ASES)[43]. Baseline viral load and CD4 counts were recorded from information obtained via eROI, participant self-upload of lab results, or screening visit. Participants were compensated $40 in the form of an Amazon gift code for their time.
3-month follow-up visit
Follow-up visits lasted one hour. Participants completed a similar survey as the one administered at baseline with the addition of measures for usability displayed to intervention participants including the Health Information Technology Usability Evaluation Scale (Health-ITUES)[44] and the post-study system usability questionnaire (PSSUQ)[45]. Participants also provided viral load and CD4 counts obtained via eROI, participant self-upload of lab results, or through DBS collection. Real time adherence data from the CleverCap pill bottles was downloaded from the CleverCap website. At the end of the follow up visit, all participants were given the option to participate in an optional qualitative interview about their experience in the study and the perceived utility of a remotely delivered CHAMPS intervention. Follow-up interviews were conducted exclusively using secure HIPAA [illegible]

Your response is too large. Try shortening some answers.

Participants were compensated $50 for their time in the follow-up visit and an additional [illegible]

$35 if they opted to participate in the interview. Compensation was provided in the form of Amazon gift codes."

4b-ii) Report how institutional affiliations are displayed

Report how institutional affiliations are displayed to potential participants [on ehealth media], as affiliations with prestigious hospitals or universities may affect volunteer rates, use, and reactions with regards to an intervention.(Not a required item – describe only if this may bias results)

	1	2	3	4	5	
subitem not at all important	○	◉	○	○	○	essential

Clear selection

Does your paper address subitem 4b-ii?

Copy and paste relevant sections from the manuscript (include quotes in quotation marks "like this" to indicate direct quotes from your manuscript), or elaborate on this item by providing additional information not in the ms, or briefly explain why the item is not applicable/relevant for your study

The e-health media used (CleverCap) did not display any insititutional affiliations as can be seen from figures 2 and 3.

5) The interventions for each group with sufficient details to allow replication, including how and when they were actually administered

Your response is too large. Try shortening some answers.

5-i) Mention names, credential, affiliations of the developers, sponsors, and owners

Mention names, credential, affiliations of the developers, sponsors, and owners [6] (if authors/evaluators are owners or developer of the software, this needs to be declared in a “Conflict of interest” section or mentioned elsewhere in the manuscript).

	1	2	3	4	5	
subitem not at all important	(•)	( )	( )	( )	( )	essential

Clear selection

Does your paper address subitem 5-i?

Copy and paste relevant sections from the manuscript (include quotes in quotation marks "like this" to indicate direct quotes from your manuscript), or elaborate on this item by providing additional information not in the ms, or briefly explain why the item is not applicable/relevant for your study

Information regarding the development (including names, credentials, and affiliations) of the application are detailed in a previous study and is thus, not relevant to our ms. (sources 34, 35, 36 in the references)

5-ii) Describe the history/development process

Describe the history/development process of the application and previous formative evaluations (e.g., focus groups, usability testing), as these will have an impact on adoption/use rates and help with interpreting results.

	1	2	3	4	5	
subitem not at all important	(•)	( )	( )	( )	( )	essential

Clear selection

Your response is too large. Try shortening some answers.

Does your paper address subitem 5-ii?

Copy and paste relevant sections from the manuscript (include quotes in quotation marks "like this" to indicate direct quotes from your manuscript), or elaborate on this item by providing additional information not in the ms, or briefly explain why the item is not applicable/relevant for your study

Information regarding the development of the application are detailed in a previous study and is thus, not relevant to our ms. (sources 34, 35, 36 in the references)

5-iii) Revisions and updating

Revisions and updating. Clearly mention the date and/or version number of the application/intervention (and comparator, if applicable) evaluated, or describe whether the intervention underwent major changes during the evaluation process, or whether the development and/or content was “frozen” during the trial. Describe dynamic components such as news feeds or changing content which may have an impact on the replicability of the intervention (for unexpected events see item 3b).

	1	2	3	4	5	
subitem not at all important	(selected)	○	○	○	○	essential

Clear selection

Does your paper address subitem 5-iii?

Copy and paste relevant sections from the manuscript (include quotes in quotation marks "like this" to indicate direct quotes from your manuscript), or elaborate on this item by providing additional information not in the ms, or briefly explain why the item is not applicable/relevant for your study

Information regarding the development of the application are detailed in a previous study and is thus, not relevant to our ms. (sources 34, 35, 36 in the references) There were no updates in our ms.

Your response is too large. Try shortening some answers.

5-iv) Quality assurance methods

Provide information on quality assurance methods to ensure accuracy and quality of information provided [1], if applicable.

	1	2	3	4	5	
subitem not at all important	(●)	( )	( )	( )	( )	essential

Clear selection

Does your paper address subitem 5-iv?

Copy and paste relevant sections from the manuscript (include quotes in quotation marks "like this" to indicate direct quotes from your manuscript), or elaborate on this item by providing additional information not in the ms, or briefly explain why the item is not applicable/relevant for your study

Information regarding the development of the application are detailed in a previous study and is thus, not relevant to our ms. (sources 34, 35, 36 in the references)

5-v) Ensure replicability by publishing the source code, and/or providing screenshots/screen-capture video, and/or providing flowcharts of the algorithms used

Ensure replicability by publishing the source code, and/or providing screenshots/screen-capture video, and/or providing flowcharts of the algorithms used. Replicability (i.e., other researchers should in principle be able to replicate the study) is a hallmark of scientific reporting.

	1	2	3	4	5	
subitem not at all important	(●)	( )	( )	( )	( )	essential

Clear selection

Your response is too large. Try shortening some answers.

Does your paper address subitem 5-v?

Copy and paste relevant sections from the manuscript (include quotes in quotation marks "like this" to indicate direct quotes from your manuscript), or elaborate on this item by providing additional information not in the ms, or briefly explain why the item is not applicable/relevant for your study

Information regarding the development of the application are detailed in a previous study and is thus, not relevant to our ms. (sources 34, 35, 36 in the references)

5-vi) Digital preservation

Digital preservation: Provide the URL of the application, but as the intervention is likely to change or disappear over the course of the years; also make sure the intervention is archived (Internet Archive, webcitation.org, and/or publishing the source code or screenshots/videos alongside the article). As pages behind login screens cannot be archived, consider creating demo pages which are accessible without login.

	1	2	3	4	5	
subitem not at all important	●	○	○	○	○	essential

Clear selection

Does your paper address subitem 5-vi?

Copy and paste relevant sections from the manuscript (include quotes in quotation marks "like this" to indicate direct quotes from your manuscript), or elaborate on this item by providing additional information not in the ms, or briefly explain why the item is not applicable/relevant for your study

Information regarding the development of the application are detailed in a previous study and is thus, not relevant to our ms. (sources 34, 35, 36 in the references). The application is open access via google playstore or the apple store.

Your response is too large. Try shortening some answers.

5-vii) Access

Access: Describe how participants accessed the application, in what setting/context, if they had to pay (or were paid) or not, whether they had to be a member of specific group. If known, describe how participants obtained “access to the platform and Internet” [1]. To ensure access for editors/reviewers/readers, consider to provide a “backdoor” login account or demo mode for reviewers/readers to explore the application (also important for archiving purposes, see vi).

	1	2	3	4	5	
subitem not at all important	○	○	○	◉	○	essential

Clear selection

Does your paper address subitem 5-vii? *

Copy and paste relevant sections from the manuscript (include quotes in quotation marks "like this" to indicate direct quotes from your manuscript), or elaborate on this item by providing additional information not in the ms, or briefly explain why the item is not applicable/relevant for your study

"Intervention participants were informed that they will be sent a CleverCap bottle and will have up to twelve study sessions over the duration of the study with an assigned CHW."

Also, in figure 1, the content of CHW session 2 implies that the study team will walk the participant on how to download the app and use it.

Your response is too large. Try shortening some answers.

5-viii) Mode of delivery, features/functionalities/components of the intervention and comparator, and the theoretical framework

Describe mode of delivery, features/functionalities/components of the intervention and comparator, and the theoretical framework [6] used to design them (instructional strategy [1], behaviour change techniques, persuasive features, etc., see e.g., [7, 8] for terminology). This includes an in-depth description of the content (including where it is coming from and who developed it) [1],” whether [and how] it is tailored to individual circumstances and allows users to track their progress and receive feedback” [6]. This also includes a description of communication delivery channels and – if computer-mediated communication is a component – whether communication was synchronous or asynchronous [6]. It also includes information on presentation strategies [1], including page design principles, average amount of text on pages, presence of hyperlinks to other resources, etc. [1].

	1	2	3	4	5	
subitem not at all important	○	○	○	◉	○	essential

Clear selection

Does your paper address subitem 5-viii? *

Copy and paste relevant sections from the manuscript (include quotes in quotation marks "like this" to indicate direct quotes from your manuscript), or elaborate on this item by providing additional information not in the ms, or briefly explain why the item is not applicable/relevant for your study

"All sessions were conducted remotely via secure HIPAA compliant Zoom calls, phone calls, or via the chat feature of the WiseApp, depending on the participant’s preference." More information is provided in the "Intervention arm and description of planned intervention" section.

Your response is too large. Try shortening some answers.

5-ix) Describe use parameters

Describe use parameters (e.g., intended “doses” and optimal timing for use). Clarify what instructions or recommendations were given to the user, e.g., regarding timing, frequency, heaviness of use, if any, or was the intervention used ad libitum.

	1	2	3	4	5	
subitem not at all important	○	○	○	○	◉	essential

Clear selection

Does your paper address subitem 5-ix?

Copy and paste relevant sections from the manuscript (include quotes in quotation marks "like this" to indicate direct quotes from your manuscript), or elaborate on this item by providing additional information not in the ms, or briefly explain why the item is not applicable/relevant for your study

"Intervention participants received one daily reminder through the CleverCap's programmed alarm to take their medication at their chosen time. Additional alarms could be programmed by participants, or mobile/app alerts could be set for missed or off-schedule doses, in which case participants received more than one daily notification. Participants were also notified if they reopened the pill bottle after an alarm had already been triggered (flagged as an off-schedule dose) or if the cap was improperly closed. However, no reminders were sent nor were any alarms triggered if the CleverCap pill bottle lost power, was deactivated by the study team, was destroyed, or had no dosing schedules set up."
More information is provided in the "Intervention arm and description of planned intervention" section.

Your response is too large. Try shortening some answers.

5-x) Clarify the level of human involvement

Clarify the level of human involvement (care providers or health professionals, also technical assistance) in the e-intervention or as co-intervention (detail number and expertise of professionals involved, if any, as well as "type of assistance offered, the timing and frequency of the support, how it is initiated, and the medium by which the assistance is delivered". It may be necessary to distinguish between the level of human involvement required for the trial, and the level of human involvement required for a routine application outside of a RCT setting (discuss under item 21 – generalizability).

	1	2	3	4	5	
subitem not at all important	○	○	○	◉	○	essential

Clear selection

Your response is too large. Try shortening some answers.

Does your paper address subitem 5-x?

Copy and paste relevant sections from the manuscript (include quotes in quotation marks "like this" to indicate direct quotes from your manuscript), or elaborate on this item by providing additional information not in the ms, or briefly explain why the item is not applicable/relevant for your study

Your response is too large. Try shortening some answers.

"The CHAMPS intervention was a three-month intervention guided by the study team's prior work on CHW and mHealth interventions. Specifically, the Birmingham Access to Care (BA2C) (NCT03205982) study adapted the Anti-Retroviral Treatment and Access to Services (ARTAS) intervention, an CDC designated evidence based intervention, to support re-engagement in care PWH who had dropped out of care[34, 35]. This approach emphasized strengths-based case management and motivational interviewing, fostering a close, supportive relationship between CHWs and participants, and was used to design the content structure of the CHW sessions. Additionally, the WiseApp study leveraged end-user feedback to develop a self-management app for PWH, incorporating features like medication trackers, push-notification reminders, and linkage to the CleverCap smart pill bottle[34, 36].

Participants in the intervention arm were assigned a CHW at the end of their baseline visit. CHWs were study team members trained on the intervention including the content of each session, motivational interviewing, strengths-based case management, ARTAS[37], HIV and substance use, the WiseApp and associated mHealth technology, and field safety. CHWs administered at least ten, but up to twelve individual sessions with the participants throughout the course of the intervention. Figure 1 provides an outline and description of CHW session content(s) and structure. All sessions were conducted remotely via secure HIPAA compliant Zoom calls, phone calls, or via the chat feature of the WiseApp, depending on the participant's preference. Intervention participants received one daily reminder through the CleverCap's programmed alarm to take their medication at their chosen time. Additional alarms could be programmed by participants, or mobile/app alerts could be set for missed or off-schedule doses, in which case participants received more than one daily notification. Participants were also notified if they reopened the pill bottle after an alarm had already been triggered (flagged as an off-schedule dose) or if the cap was improperly closed. However, no reminders were sent nor were any alarms triggered if the CleverCap pill bottle lost power, was deactivated by the study team, was destroyed, or had no dosing schedules set up.

Figure 2 illustrates the WiseApp user interface, with screenshots of a demo account. The main menu (Figure 2A) allows navigation to various tabs. The dashboard (Figure 2B) provides an overview of medication adherence, including statuses such as taken (green), missed (red), off-schedule dose (yellow), and improperly closed cap (orange). It also displays adherence feedback through emoji indicators (e.g., high adherence = green smiley face). The chat interface (Figure 2C) enables communication with CHWs, while the 'My Stats' tab (Figure 2D) presents a percentage breakdown of adherence metrics, dose timing, and adherence scores. The 'Videos and Information' tab (Figure 2E) includes testimonial videos with adherence tips. The 'My Alerts' tab (Figure 2F) allows participants and CHWs to set reminders for missed or off-schedule doses. Finally, the 'My Meds' tab (Figure 2G) enables participants and CHWs to manage medication details and adjust dosing schedules.

The CleverCap pill bottle (Figure 3) tracked medication adherence by recording when the bottle was opened, with a dose marked as "Taken" only if the cap remained off for at least five seconds. Figure 3A shows the CleverCap and its packaging, which included a micro-USB charger. Figure 3B demonstrates the CleverCap's built-in reminder system, which provided a visual and auditory alarm when it was time to take a scheduled dose. The bottle flashed green lights for two minutes and emitted a loud alarm to ensure the reminder was [illegible]

Your response is too large. Try shortening some answers.

[illegible] the visual and auditory cues stopped, and the dose was recorded as 'Missed.' No visual or [illegible]

auditory cues were provided for off-schedule doses. If the alarm failed to activate due to incorrect or missing programmed alerts on the WiseApp or due to the pill bottle losing power, participants may have missed their medication without documentation on the app."

5-xi) Report any prompts/reminders used

Report any prompts/reminders used: Clarify if there were prompts (letters, emails, phone calls, SMS) to use the application, what triggered them, frequency etc. It may be necessary to distinguish between the level of prompts/reminders required for the trial, and the level of prompts/reminders for a routine application outside of a RCT setting (discuss under item 21 – generalizability).

	1	2	3	4	5	
subitem not at all important	○	○	○	○	◉	essential

Clear selection

Your response is too large. Try shortening some answers.

Does your paper address subitem 5-xi? *

Copy and paste relevant sections from the manuscript (include quotes in quotation marks "like this" to indicate direct quotes from your manuscript), or elaborate on this item by providing additional information not in the ms, or briefly explain why the item is not applicable/relevant for your study

"Intervention participants received one daily reminder through the CleverCap's programmed alarm to take their medication at their chosen time. Additional alarms could be programmed by participants, or mobile/app alerts could be set for missed or off-schedule doses, in which case participants received more than one daily notification. Participants were also notified if they reopened the pill bottle after an alarm had already been triggered (flagged as an off-schedule dose) or if the cap was improperly closed. However, no reminders were sent nor were any alarms triggered if the CleverCap pill bottle lost power, was deactivated by the study team, was destroyed, or had no dosing schedules set up.
Figure 2 illustrates the WiseApp user interface, with screenshots of a demo account. The main menu (Figure 2A) allows navigation to various tabs. The dashboard (Figure 2B) provides an overview of medication adherence, including statuses such as taken (green), missed (red), off-schedule dose (yellow), and improperly closed cap (orange). It also displays adherence feedback through emoji indicators (e.g., high adherence = green smiley face). The chat interface (Figure 2C) enables communication with CHWs, while the 'My Stats' tab (Figure 2D) presents a percentage breakdown of adherence metrics, dose timing, and adherence scores. The 'Videos and Information' tab (Figure 2E) includes testimonial videos with adherence tips. The 'My Alerts' tab (Figure 2F) allows participants and CHWs to set reminders for missed or off-schedule doses. Finally, the 'My Meds' tab (Figure 2G) enables participants and CHWs to manage medication details and adjust dosing schedules.
The CleverCap pill bottle (Figure 3) tracked medication adherence by recording when the bottle was opened, with a dose marked as "Taken" only if the cap remained off for at least five seconds. Figure 3A shows the CleverCap and its packaging, which included a micro-USB charger. Figure 3B demonstrates the CleverCap's built-in reminder system, which provided a visual and auditory alarm when it was time to take a scheduled dose. The bottle flashed green lights for two minutes and emitted a loud alarm to ensure the reminder was noticeable. These cues automatically ceased once the cap was unscrewed, signaling a recorded dose. Additionally, if no action was taken within the two-minute reminder window, the visual and auditory cues stopped, and the dose was recorded as 'Missed.' No visual or auditory cues were provided for off-schedule doses. If the alarm failed to activate due to incorrect or missing programmed alerts on the WiseApp or due to the pill bottle losing power, participants may have missed their medication without documentation on the app."

Your response is too large. Try shortening some answers.

5-xii) Describe any co-interventions (incl. training/support)

Describe any co-interventions (incl. training/support): Clearly state any interventions that are provided in addition to the targeted eHealth intervention, as ehealth intervention may not be designed as stand-alone intervention. This includes training sessions and support [1]. It may be necessary to distinguish between the level of training required for the trial, and the level of training for a routine application outside of a RCT setting (discuss under item 21 – generalizability.

	1	2	3	4	5	
subitem not at all important	○	○	○	○	◉	essential

Clear selection

Does your paper address subitem 5-xii? *

Copy and paste relevant sections from the manuscript (include quotes in quotation marks "like this" to indicate direct quotes from your manuscript), or elaborate on this item by providing additional information not in the ms, or briefly explain why the item is not applicable/relevant for your study

"CHWs were study team members trained on the intervention including the content of each session, motivational interviewing, strengths-based case management, ARTAS[37], HIV and substance use, the WiseApp and associated mHealth technology, and field safety. CHWs administered at least ten, but up to twelve individual sessions with the participants throughout the course of the intervention. Figure 1 provides an outline and description of CHW session content(s) and structure."

6a) Completely defined pre-specified primary and secondary outcome measures, including how and when they were assessed

Your response is too large. Try shortening some answers.

Does your paper address CONSORT subitem 6a? *

Copy and paste relevant sections from the manuscript (include quotes in quotation marks "like this" to indicate direct quotes from your manuscript), or elaborate on this item by providing additional information not in the ms, or briefly explain why the item is not applicable/relevant for your study

"The primary outcome, viral load (virally unsuppressed vs. suppressed), was analyzed using a two-way frequency table by time. The adjusted odds ratio (aOR), adjusting for baseline viral load status, was calculated for the CHAMPS group (intervention vs. control).
Self-rated adherence scores using the SRSI[42], a 6-point Likert scale ranging from 1 ("Very Poor") to 6 ("Excellent"), were reported as mean and standard deviation (SD). Independent t-tests were used to compare self-rated adherence scores between the intervention and control groups, while paired t-tests were used to compare scores within groups over time. Missing data for the paired t-test was handled under the assumption that it was missing completely at random (MCAR), as participants were lost to follow-up due to external factors unrelated to study data collection.
For adherence data collected through CleverCap, the average percentage of doses taken and missed were calculated with SD reported for the analyzed intervention group participants. The correlation between follow-up SRSI scores and the average percentage of doses taken was calculated using Pearson’s correlation.
Usability measures, including the PSSUQ [45] and the Health-ITUES[44], were analyzed by calculating the mean and SD for the overall scales and their subscales. The PSSUQ (18 items, 3 subscales) was measured using a 7-point Likert scale, ranging from 1 ("Strongly Agree") to 7 ("Strongly Disagree"). The Health-ITUES (20 items, 4 subscales) was measured using a 5-point Likert scale, ranging from 1 ("Strongly Disagree") to 5 ("Strongly Agree").
Self-efficacy for HIV treatment adherence was measured using the HIV-ASES scale (12 items, 2 subscales)[43], a 10-point Likert scale ranging from 1 ("Cannot do at all") to 10 ("Certain can do it"). Mean and SD were computed for self-efficacy scores. Independent t-tests were used to compare HIV-ASES scores between groups, and paired t-tests were used to compare within groups over time, with missing data handled as MCAR due to external factors unrelated to the study..."
"An initial codebook was developed based on the Mobile Health Technology Acceptance Model (MHTAM), which includes technological, individual, and social factors that influence the acceptance of healthcare technology[22]. This model has been adapted for this study to examine an individual’s intention to use mHealth interventions for HIV treatment adherence or participate in the CHAMPS Pilot study procedures as it examines key factors such as perceived usefulness, ease of use, and ubiquity, alongside individual beliefs and social influences (Figure 4)."

Your response is too large. Try shortening some answers.

6a-i) Online questionnaires: describe if they were validated for online use and apply CHERRIES items to describe how the questionnaires were designed/deployed

If outcomes were obtained through online questionnaires, describe if they were validated for online use and apply CHERRIES items to describe how the questionnaires were designed/deployed [9].

	1	2	3	4	5	
subitem not at all important	(•)	( )	( )	( )	( )	essential

Clear selection

Does your paper address subitem 6a-i?

Copy and paste relevant sections from manuscript text

N/A did not use existing online questionnaires.

6a-ii) Describe whether and how "use" (including intensity of use/dosage) was defined/measured/monitored

Describe whether and how "use" (including intensity of use/dosage) was defined/measured/monitored (logins, logfile analysis, etc.). Use/adoption metrics are important process outcomes that should be reported in any ehealth trial.

	1	2	3	4	5	
subitem not at all important	( )	( )	(•)	( )	( )	essential

Clear selection

Your response is too large. Try shortening some answers.

Does your paper address subitem 6a-ii?

Copy and paste relevant sections from manuscript text

"For adherence data collected through CleverCap, the average percentage of doses taken and missed were calculated with SD reported for the analyzed intervention group participants. The correlation between follow-up SRSI scores and the average percentage of doses taken was calculated using Pearson's correlation."

6a-iii) Describe whether, how, and when qualitative feedback from participants was obtained

Describe whether, how, and when qualitative feedback from participants was obtained (e.g., through emails, feedback forms, interviews, focus groups).

	1	2	3	4	5	
subitem not at all important	○	○	○	○	◉	essential

Clear selection

Does your paper address subitem 6a-iii?

Copy and paste relevant sections from manuscript text

"At the end of the follow up visit, all participants were given the option to participate in an optional qualitative interview about their experience in the study and the perceived utility of a remotely delivered CHAMPS intervention. Follow-up interviews were conducted exclusively using secure HIPAA compliant Zoom technology and were audio-recorded for transcription. Consent for follow-up interviews was included in the study consent offered to participants at the baseline visit. Participants were compensated $50 for their time in the follow-up visit and an additional $35 if they opted to participate in the interview. Compensation was provided in the form of Amazon gift codes."

6b) Any changes to trial outcomes after the trial commenced, with reasons

Your response is too large. Try shortening some answers.

Does your paper address CONSORT subitem 6b? *

Copy and paste relevant sections from the manuscript (include quotes in quotation marks "like this" to indicate direct quotes from your manuscript), or elaborate on this item by providing additional information not in the ms, or briefly explain why the item is not applicable/relevant for your study

N/A no changes were made to trial outcomes after the trial commenced.

7a) How sample size was determined

NPT: When applicable, details of whether and how the clustering by care provides or centers was addressed

7a-i) Describe whether and how expected attrition was taken into account when calculating the sample size

Describe whether and how expected attrition was taken into account when calculating the sample size.

	1	2	3	4	5	
subitem not at all important	(●)	○	○	○	○	essential

Clear selection

Does your paper address subitem 7a-i?

Copy and paste relevant sections from manuscript title (include quotes in quotation marks "like this" to indicate direct quotes from your manuscript), or elaborate on this item by providing additional information not in the ms, or briefly explain why the item is not applicable/relevant for your study

Sample size calculation is detailed below based on the protocol but reporting was deemed unessential as this is a pilot study aimed at determining feasibility.

Your response is too large. Try shortening some answers.

7b) When applicable, explanation of any interim analyses and stopping guidelines

Does your paper address CONSORT subitem 7b? *

Copy and paste relevant sections from the manuscript (include quotes in quotation marks "like this" to indicate direct quotes from your manuscript), or elaborate on this item by providing additional information not in the ms, or briefly explain why the item is not applicable/relevant for your study

N/A not applicable as there were no interim analyzes or stopping guidelines required - see above.

8a) Method used to generate the random allocation sequence
NPT: When applicable, how care providers were allocated to each trial group

Does your paper address CONSORT subitem 8a? *

Copy and paste relevant sections from the manuscript (include quotes in quotation marks "like this" to indicate direct quotes from your manuscript), or elaborate on this item by providing additional information not in the ms, or briefly explain why the item is not applicable/relevant for your study

"Upon enrollment, participants were randomized to either intervention or control arms through a randomization module in REDCap. "

8b) Type of randomisation; details of any restriction (such as blocking and block size)

Your response is too large. Try shortening some answers.

Does your paper address CONSORT subitem 8b? *

Copy and paste relevant sections from the manuscript (include quotes in quotation marks "like this" to indicate direct quotes from your manuscript), or elaborate on this item by providing additional information not in the ms, or briefly explain why the item is not applicable/relevant for your study

"Upon enrollment, participants were randomized to either intervention or control arms through a randomization module in REDCap."

there were no blocks required as this was a 1:1 allocation.

9) Mechanism used to implement the random allocation sequence (such as sequentially numbered containers), describing any steps taken to conceal the sequence until interventions were assigned

Does your paper address CONSORT subitem 9? *

Copy and paste relevant sections from the manuscript (include quotes in quotation marks "like this" to indicate direct quotes from your manuscript), or elaborate on this item by providing additional information not in the ms, or briefly explain why the item is not applicable/relevant for your study

"Upon enrollment, participants were randomized to either intervention or control arms through a randomization module in REDCap."

10) Who generated the random allocation sequence, who enrolled participants, and who assigned participants to interventions

Your response is too large. Try shortening some answers.

Does your paper address CONSORT subitem 10? *

Copy and paste relevant sections from the manuscript (include quotes in quotation marks "like this" to indicate direct quotes from your manuscript), or elaborate on this item by providing additional information not in the ms, or briefly explain why the item is not applicable/relevant for your study

"The randomization module was generated by the study team's data manager, ensuring an unbiased allocation process, while study staff - who were separate from the data manager and did not have access to the randomization module - were responsible for assigning participants to their respective study groups."

11a) If done, who was blinded after assignment to interventions (for example, participants, care providers, those assessing outcomes) and how

NPT: Whether or not administering co-interventions were blinded to group assignment

11a-i) Specify who was blinded, and who wasn't

Specify who was blinded, and who wasn't. Usually, in web-based trials it is not possible to blind the participants [1, 3] (this should be clearly acknowledged), but it may be possible to blind outcome assessors, those doing data analysis or those administering co-interventions (if any).

	1	2	3	4	5	
subitem not at all important	○	○	○	◉	○	essential

Clear selection

Your response is too large. Try shortening some answers.

Does your paper address subitem 11a-i? *

Copy and paste relevant sections from the manuscript (include quotes in quotation marks "like this" to indicate direct quotes from your manuscript), or elaborate on this item by providing additional information not in the ms, or briefly explain why the item is not applicable/relevant for your study

This was an unblinded study - "while study staff - who were separate from the data manager and did not have access to the randomization module - were responsible for assigning participants to their respective study groups. Intervention participants were informed that they will be sent a CleverCap bottle and will have up to twelve study sessions over the duration of the study with an assigned CHW." The study staff had to know the participants were in the intervention group to ship the pill bottle and assign CHWs. Thus the study participants also knew of their status.

11a-ii) Discuss e.g., whether participants knew which intervention was the “intervention of interest” and which one was the “comparator”

Informed consent procedures (4a-ii) can create biases and certain expectations - discuss e.g., whether participants knew which intervention was the “intervention of interest” and which one was the “comparator”.

	1	2	3	4	5	
subitem not at all important	○	○	○	○	◉	essential

Clear selection

Your response is too large. Try shortening some answers.

Does your paper address subitem 11a-ii?

Copy and paste relevant sections from the manuscript (include quotes in quotation marks "like this" to indicate direct quotes from your manuscript), or elaborate on this item by providing additional information not in the ms, or briefly explain why the item is not applicable/relevant for your study

This was an unblinded study - "while study staff - who were separate from the data manager and did not have access to the randomization module - were responsible for assigning participants to their respective study groups. Intervention participants were informed that they will be sent a CleverCap bottle and will have up to twelve study sessions over the duration of the study with an assigned CHW." The study staff had to know the participants were in the intervention group to ship the pill bottle and assign CHWs. Thus the study participants also knew of their status.

11b) If relevant, description of the similarity of interventions

(this item is usually not relevant for ehealth trials as it refers to similarity of a placebo or sham intervention to a active medication/intervention)

Does your paper address CONSORT subitem 11b? *

Copy and paste relevant sections from the manuscript (include quotes in quotation marks "like this" to indicate direct quotes from your manuscript), or elaborate on this item by providing additional information not in the ms, or briefly explain why the item is not applicable/relevant for your study

N/A - the control group got standard of care without the pill bottle or the CHW sessions.

12a) Statistical methods used to compare groups for primary and secondary outcomes

NPT: When applicable, details of whether and how the clustering by care providers or centers was addressed

Your response is too large. Try shortening some answers.

Does your paper address CONSORT subitem 12a? *

Copy and paste relevant sections from the manuscript (include quotes in quotation marks "like this" to indicate direct quotes from your manuscript), or elaborate on this item by providing additional information not in the ms, or briefly explain why the item is not applicable/relevant for your study

"Descriptive statistics for demographic variables were reported as counts and percentages. Statistical significance for differences between the control and intervention groups was assessed using the Mann-Whitney U test for continuous variables, such as ages, and the chi-squared test for the remaining categorical variables. A two-sample z-test for equality of proportions, with a continuity correction, was used to assess whether the differences in retention rates between the intervention and control groups were statistically significant.
The primary outcome, viral load (virally unsuppressed vs. suppressed), was analyzed using a two-way frequency table by time. The adjusted odds ratio (aOR), adjusting for baseline viral load status, was calculated for the CHAMPS group (intervention vs. control).
Self-rated adherence scores using the SRSI[42], a 6-point Likert scale ranging from 1 ("Very Poor") to 6 ("Excellent"), were reported as mean and standard deviation (SD). Independent t-tests were used to compare self-rated adherence scores between the intervention and control groups, while paired t-tests were used to compare scores within groups over time. Missing data for the paired t-test was handled under the assumption that it was missing completely at random (MCAR), as participants were lost to follow-up due to external factors unrelated to study data collection.
For adherence data collected through CleverCap, the average percentage of doses taken and missed were calculated with SD reported for the analyzed intervention group participants. The correlation between follow-up SRSI scores and the average percentage of doses taken was calculated using Pearson’s correlation.
Usability measures, including the PSSUQ [45] and the Health-ITUES[44], were analyzed by calculating the mean and SD for the overall scales and their subscales. The PSSUQ (18 items, 3 subscales) was measured using a 7-point Likert scale, ranging from 1 ("Strongly Agree") to 7 ("Strongly Disagree"). The Health-ITUES (20 items, 4 subscales) was measured using a 5-point Likert scale, ranging from 1 ("Strongly Disagree") to 5 ("Strongly Agree").
Self-efficacy for HIV treatment adherence was measured using the HIV-ASES scale (12 items, 2 subscales)[43], a 10-point Likert scale ranging from 1 ("Cannot do at all") to 10 ("Certain can do it"). Mean and SD were computed for self-efficacy scores. Independent t-tests were used to compare HIV-ASES scores between groups, and paired t-tests were used to compare within groups over time, with missing data handled as MCAR due to external factors unrelated to the study.
Cronbach's alpha was calculated for the SRSI, Health-ITUES, PSSUQ, and HIV-ASES scales to measure internal reliability. Statistical analyses were conducted using SAS [46] and R software[47], and significance was determined at a p-value of 0.05."

Your response is too large. Try shortening some answers.

12a-i) Imputation techniques to deal with attrition / missing values

Imputation techniques to deal with attrition / missing values: Not all participants will use the intervention/comparator as intended and attrition is typically high in ehealth trials. Specify how participants who did not use the application or dropped out from the trial were treated in the statistical analysis (a complete case analysis is strongly discouraged, and simple imputation techniques such as LOCF may also be problematic [4]).

	1	2	3	4	5	
subitem not at all important	○	○	○	○	◉	essential

Clear selection

Does your paper address subitem 12a-i? *

Copy and paste relevant sections from the manuscript (include quotes in quotation marks "like this" to indicate direct quotes from your manuscript), or elaborate on this item by providing additional information not in the ms, or briefly explain why the item is not applicable/relevant for your study

"Missing data for the paired t-test was handled under the assumption that it was missing completely at random (MCAR), as participants were lost to follow-up due to external factors unrelated to study data collection."

12b) Methods for additional analyses, such as subgroup analyses and adjusted analyses

Does your paper address CONSORT subitem 12b? *

Copy and paste relevant sections from the manuscript (include quotes in quotation marks "like this" to indicate direct quotes from your manuscript), or elaborate on this item by providing additional information not in the ms, or briefly explain why the item is not applicable/relevant for your study

There were no planned subgroup analyses

Your response is too large. Try shortening some answers.

X26) REB/IRB Approval and Ethical Considerations [recommended as subheading under "Methods"] (not a CONSORT item)

X26-i) Comment on ethics committee approval

	1	2	3	4	5	
subitem not at all important	○	○	○	○	◉	essential

Clear selection

Does your paper address subitem X26-i?

Copy and paste relevant sections from the manuscript (include quotes in quotation marks "like this" to indicate direct quotes from your manuscript), or elaborate on this item by providing additional information not in the ms, or briefly explain why the item is not applicable/relevant for your study

"All study procedures were reviewed and approved by the Columbia University Institutional Review Board [Protocol Number AAAU2064] prior to the recruitment or enrollment of participants."

x26-ii) Outline informed consent procedures

Outline informed consent procedures e.g., if consent was obtained offline or online (how? Checkbox, etc.?), and what information was provided (see 4a-ii). See [6] for some items to be included in informed consent documents.

	1	2	3	4	5	
subitem not at all important	○	○	○	○	◉	essential

Clear selection

Your response is too large. Try shortening some answers.

Does your paper address subitem X26-ii?

Copy and paste relevant sections from the manuscript (include quotes in quotation marks "like this" to indicate direct quotes from your manuscript), or elaborate on this item by providing additional information not in the ms, or briefly explain why the item is not applicable/relevant for your study

"Participants provided electronic written consent, including a screening consent for those requiring a screening visit and a study consent for those proceeding directly to baseline. The baseline consent included language informing participants of an optional follow-up interview, and participants could indicate their willingness to participate by initialing the consent form. Study data, including interview transcripts, were anonymized and de-identified to ensure confidentiality. Participants were compensated as follows: no compensation for the screening visit, $40 for the baseline visit, $50 for the follow-up visit, and $35 for the optional qualitative interview at the follow-up visit. All images and data presented in this manuscript are de-identified to ensure privacy of our participants."

X26-iii) Safety and security procedures

Safety and security procedures, incl. privacy considerations, and any steps taken to reduce the likelihood or detection of harm (e.g., education and training, availability of a hotline)

	1	2	3	4	5	
subitem not at all important	○	○	○	○	◉	essential

Clear selection

Does your paper address subitem X26-iii?

Copy and paste relevant sections from the manuscript (include quotes in quotation marks "like this" to indicate direct quotes from your manuscript), or elaborate on this item by providing additional information not in the ms, or briefly explain why the item is not applicable/relevant for your study

"Study data, including interview transcripts, were anonymized and de-identified to ensure confidentiality. All images and data presented in this manuscript are de-identified to ensure privacy of our participants."

Your response is too large. Try shortening some answers.

RESULTS

13a) For each group, the numbers of participants who were randomly assigned, received intended treatment, and were analysed for the primary outcome

NPT: The number of care providers or centers performing the intervention in each group and the number of patients treated by each care provider in each center

Does your paper address CONSORT subitem 13a? *

Copy and paste relevant sections from the manuscript (include quotes in quotation marks "like this" to indicate direct quotes from your manuscript), or elaborate on this item by providing additional information not in the ms, or briefly explain why the item is not applicable/relevant for your study

Yes, please see figure 5.

13b) For each group, losses and exclusions after randomisation, together with reasons

Does your paper address CONSORT subitem 13b? (NOTE: Preferably, this is shown in a CONSORT flow diagram) *

Copy and paste relevant sections from the manuscript (include quotes in quotation marks "like this" to indicate direct quotes from your manuscript), or elaborate on this item by providing additional information not in the ms, or briefly explain why the item is not applicable/relevant for your study

Yes, please see figure 5.

Your response is too large. Try shortening some answers.

13b-i) Attrition diagram

Strongly recommended: An attrition diagram (e.g., proportion of participants still logging in or using the intervention/comparator in each group plotted over time, similar to a survival curve) or other figures or tables demonstrating usage/dose/engagement.

	1	2	3	4	5	
subitem not at all important	○	◉	○	○	○	essential

Clear selection

Does your paper address subitem 13b-i?

Copy and paste relevant sections from the manuscript or cite the figure number if applicable (include quotes in quotation marks "like this" to indicate direct quotes from your manuscript), or elaborate on this item by providing additional information not in the ms, or briefly explain why the item is not applicable/relevant for your study

N/A While we do not have an attrition diagram as reporting at an individual level offers the risk of de-identification. Instead we have group level data on attrition presented as a table (see table 3). Of relevance, readers should pay attention to the # of missed doses or off-schedule doses.

14a) Dates defining the periods of recruitment and follow-up

Does your paper address CONSORT subitem 14a? *

Copy and paste relevant sections from the manuscript (include quotes in quotation marks "like this" to indicate direct quotes from your manuscript), or elaborate on this item by providing additional information not in the ms, or briefly explain why the item is not applicable/relevant for your study

"Recruitment for the study occurred over a seven-month period, beginning in July 2023 and closing at the end of January 2024."

Your response is too large. Try shortening some answers.

14a-i) Indicate if critical “secular events” fell into the study period

Indicate if critical “secular events” fell into the study period, e.g., significant changes in Internet resources available or “changes in computer hardware or Internet delivery resources”

	1	2	3	4	5	
subitem not at all important	(•)	○	○	○	○	essential

Clear selection

Does your paper address subitem 14a-i?

Copy and paste relevant sections from the manuscript (include quotes in quotation marks "like this" to indicate direct quotes from your manuscript), or elaborate on this item by providing additional information not in the ms, or briefly explain why the item is not applicable/relevant for your study

There were no secular periods during our recruitment period.

14b) Why the trial ended or was stopped (early)

Does your paper address CONSORT subitem 14b? *

Copy and paste relevant sections from the manuscript (include quotes in quotation marks "like this" to indicate direct quotes from your manuscript), or elaborate on this item by providing additional information not in the ms, or briefly explain why the item is not applicable/relevant for your study

N/A the trial was not stopped early.

Your response is too large. Try shortening some answers.

15) A table showing baseline demographic and clinical characteristics for each group

NPT: When applicable, a description of care providers (case volume, qualification, expertise, etc.) and centers (volume) in each group

Does your paper address CONSORT subitem 15? *

Copy and paste relevant sections from the manuscript (include quotes in quotation marks "like this" to indicate direct quotes from your manuscript), or elaborate on this item by providing additional information not in the ms, or briefly explain why the item is not applicable/relevant for your study

Yes, please see table 1 - Baseline sociodemographic characteristics of participants, comparing the control and intervention groups

15-i) Report demographics associated with digital divide issues

In ehealth trials it is particularly important to report demographics associated with digital divide issues, such as age, education, gender, social-economic status, computer/Internet/ehealth literacy of the participants, if known.

	1	2	3	4	5	
subitem not at all important	○	○	○	○	◉	essential

Clear selection

Does your paper address subitem 15-i? *

Copy and paste relevant sections from the manuscript (include quotes in quotation marks "like this" to indicate direct quotes from your manuscript), or elaborate on this item by providing additional information not in the ms, or briefly explain why the item is not applicable/relevant for your study

All the above demographics are reported in table 1.

Your response is too large. Try shortening some answers.

16) For each group, number of participants (denominator) included in each analysis and whether the analysis was by original assigned groups

16-i) Report multiple “denominators” and provide definitions

Report multiple “denominators” and provide definitions: Report N’s (and effect sizes) “across a range of study participation [and use] thresholds” [1], e.g., N exposed, N consented, N used more than x times, N used more than y weeks, N participants “used” the intervention/comparator at specific pre-defined time points of interest (in absolute and relative numbers per group). Always clearly define “use” of the intervention.

	1	2	3	4	5	
subitem not at all important	○	○	○	○	◉	essential

Clear selection

Does your paper address subitem 16-i? *

Copy and paste relevant sections from the manuscript (include quotes in quotation marks "like this" to indicate direct quotes from your manuscript), or elaborate on this item by providing additional information not in the ms, or briefly explain why the item is not applicable/relevant for your study

Here is an example of such reporting: "At baseline, the control group (n = 20) had a mean HIV-ASES Overall score of 8.24 (SD ± 1.96), and the intervention group (n = 20) had a mean score of 8.08 (SD ± 2.25). At follow-up, the control group’s (n = 18) score increased to 9.06 (SD ± 1.08), while the intervention group’s (n = 12) score was 8.78 (SD ± 2.16). Although unexpected, the intervention group's lower self-efficacy score compared to the control group may reflect increased awareness of their adherence habits through engagement with the CHAMPS intervention, leading to more accurate and self-critical reporting."

Your response is too large. Try shortening some answers.

16-ii) Primary analysis should be intent-to-treat

Primary analysis should be intent-to-treat, secondary analyses could include comparing only “users”, with the appropriate caveats that this is no longer a randomized sample (see 18-i).

	1	2	3	4	5	
subitem not at all important	○	○	◉	○	○	essential

Clear selection

Does your paper address subitem 16-ii?

Copy and paste relevant sections from the manuscript (include quotes in quotation marks "like this" to indicate direct quotes from your manuscript), or elaborate on this item by providing additional information not in the ms, or briefly explain why the item is not applicable/relevant for your study

Our analysis uses intent-to-treat analysis primarily for baseline values where we have complete data. We employ a users only approach for follow-up specific data.

17a) For each primary and secondary outcome, results for each group, and the estimated effect size and its precision (such as 95% confidence interval)

Does your paper address CONSORT subitem 17a? *

Copy and paste relevant sections from the manuscript (include quotes in quotation marks "like this" to indicate direct quotes from your manuscript), or elaborate on this item by providing additional information not in the ms, or briefly explain why the item is not applicable/relevant for your study

Yes, the results are presented with appropriate effect sizes.

Your response is too large. Try shortening some answers.

17a-i) Presentation of process outcomes such as metrics of use and intensity of use

In addition to primary/secondary (clinical) outcomes, the presentation of process outcomes such as metrics of use and intensity of use (dose, exposure) and their operational definitions is critical. This does not only refer to metrics of attrition (13-b) (often a binary variable), but also to more continuous exposure metrics such as “average session length”. These must be accompanied by a technical description how a metric like a “session” is defined (e.g., timeout after idle time) [1] (report under item 6a).

	1	2	3	4	5	
subitem not at all important	○	○	○	◉	○	essential

Clear selection

Does your paper address subitem 17a-i?

Copy and paste relevant sections from the manuscript (include quotes in quotation marks "like this" to indicate direct quotes from your manuscript), or elaborate on this item by providing additional information not in the ms, or briefly explain why the item is not applicable/relevant for your study

here is an example "A total of 10 CHW sessions were conducted as planned, though the optional two additional sessions (making it "up to 12") were not utilized due to declining participant engagement and the proximity of some sessions to participants' final follow-up visits. CHWs used HIPAA-compliant Zoom calls, phone calls, and the WiseApp chat feature based on participant preferences. Completion rates (Table 2) were initially high, with 17 out of the 20 intervention participants (85%) completing Sessions 1–3, but this declined to 9 participants (45%) by the tenth session. Participants generally preferred phone calls for session delivery, with a consistent majority opting for this modality across all sessions. The variability in delivery modes during Sessions 1 and 2 reflects logistical factors: some participants had not yet received their CleverCap devices at the time of their baseline visit, necessitating follow-up sessions via Zoom or phone for Session 2. Additionally, three participants disengaged from the study immediately after the baseline visit, which contributed to the drop-in session completion rates early in the intervention. To re-engage participants who missed sessions, CHWs attempted up to three follow-ups using participants' disclosed communication preferences (calls, texts, or emails). When no response was received after three attempts, re-engagement efforts were discontinued."

Your response is too large. Try shortening some answers.

17b) For binary outcomes, presentation of both absolute and relative effect sizes is recommended

Does your paper address CONSORT subitem 17b? *

Copy and paste relevant sections from the manuscript (include quotes in quotation marks "like this" to indicate direct quotes from your manuscript), or elaborate on this item by providing additional information not in the ms, or briefly explain why the item is not applicable/relevant for your study

Please see table 4 and accompanying narrative, where in which we report a binary outcome with a relative effect size to compare baseline variability in viral load status. Absolute effect sizes would not be beneficial in this case as it would not control for baseline variability in viral load status between study arms.

18) Results of any other analyses performed, including subgroup analyses and adjusted analyses, distinguishing pre-specified from exploratory

Does your paper address CONSORT subitem 18? *

Copy and paste relevant sections from the manuscript (include quotes in quotation marks "like this" to indicate direct quotes from your manuscript), or elaborate on this item by providing additional information not in the ms, or briefly explain why the item is not applicable/relevant for your study

N/A no subgroup analysis was conducted.

Your response is too large. Try shortening some answers.

18-i) Subgroup analysis of comparing only users

A subgroup analysis of comparing only users is not uncommon in ehealth trials, but if done, it must be stressed that this is a self-selected sample and no longer an unbiased sample from a randomized trial (see 16-iii).

	1	2	3	4	5	
subitem not at all important	◉	○	○	○	○	essential

Clear selection

Does your paper address subitem 18-i?

Copy and paste relevant sections from the manuscript (include quotes in quotation marks "like this" to indicate direct quotes from your manuscript), or elaborate on this item by providing additional information not in the ms, or briefly explain why the item is not applicable/relevant for your study

N/A no subgroup analysis was conducted.

19) All important harms or unintended effects in each group

(for specific guidance see CONSORT for harms)

Does your paper address CONSORT subitem 19? *

Copy and paste relevant sections from the manuscript (include quotes in quotation marks "like this" to indicate direct quotes from your manuscript), or elaborate on this item by providing additional information not in the ms, or briefly explain why the item is not applicable/relevant for your study

No unintended harms were reported.

Your response is too large. Try shortening some answers.

19-i) Include privacy breaches, technical problems

Include privacy breaches, technical problems. This does not only include physical "harm" to participants, but also incidents such as perceived or real privacy breaches [1], technical problems, and other unexpected/unintended incidents. "Unintended effects" also includes unintended positive effects [2].

	1	2	3	4	5	
subitem not at all important	(selected)					essential

Clear selection

Does your paper address subitem 19-i?

Copy and paste relevant sections from the manuscript (include quotes in quotation marks "like this" to indicate direct quotes from your manuscript), or elaborate on this item by providing additional information not in the ms, or briefly explain why the item is not applicable/relevant for your study

No unintended harms were reported.

19-ii) Include qualitative feedback from participants or observations from staff/researchers

Include qualitative feedback from participants or observations from staff/researchers, if available, on strengths and shortcomings of the application, especially if they point to unintended/unexpected effects or uses. This includes (if available) reasons for why people did or did not use the application as intended by the developers.

	1	2	3	4	5	
subitem not at all important					(selected)	essential

Clear selection

Your response is too large. Try shortening some answers.

Does your paper address subitem 19-ii?

Copy and paste relevant sections from the manuscript (include quotes in quotation marks "like this" to indicate direct quotes from your manuscript), or elaborate on this item by providing additional information not in the ms, or briefly explain why the item is not applicable/relevant for your study

Example: "Feedback from participants can help describe the mismatch in self-reported scores and actual adherence as individual factors, such as personal preferences in medication management, were identified as mediators of adherence. One participant noted, "I know that Biktarvy has a 50-hour shelf-life. And so, I'm not really stressing over when I take it. I usually just make sure I take it before lunch." (CHP02)" Qualitative feedback is woven throughout the ms.

DISCUSSION

22) Interpretation consistent with results, balancing benefits and harms, and considering other relevant evidence

NPT: In addition, take into account the choice of the comparator, lack of or partial blinding, and unequal expertise of care providers or centers in each group

22-i) Restate study questions and summarize the answers suggested by the data, starting with primary outcomes and process outcomes (use)

Restate study questions and summarize the answers suggested by the data, starting with primary outcomes and process outcomes (use).

	1	2	3	4	5	
subitem not at all important	○	○	○	○	◉	essential

Clear selection

Your response is too large. Try shortening some answers.

Does your paper address subitem 22-i? *

Copy and paste relevant sections from the manuscript (include quotes in quotation marks "like this" to indicate direct quotes from your manuscript), or elaborate on this item by providing additional information not in the ms, or briefly explain why the item is not applicable/relevant for your study

"This pilot study aimed to primarily assess the feasibility and usability of a remotely delivered CHAMPS intervention. While the preliminary results did not demonstrate significant improvements in adherence or self-efficacy in the intervention group compared to the control group, the findings provide important insights into how participant attitudes to use the intervention were shaped by technological, individual, and social factors."

22-ii) Highlight unanswered new questions, suggest future research

Highlight unanswered new questions, suggest future research.

	1	2	3	4	5	
subitem not at all important	○	○	○	◉	○	essential

Clear selection

Your response is too large. Try shortening some answers.

Does your paper address subitem 22-ii?

Copy and paste relevant sections from the manuscript (include quotes in quotation marks "like this" to indicate direct quotes from your manuscript), or elaborate on this item by providing additional information not in the ms, or briefly explain why the item is not applicable/relevant for your study

"Thus, understanding these sociodemographic factors is essential for contextualizing the intervention's feasibility and the challenges participants faced in engaging with CHAMPS. The demographic characteristics of participants in this study partially align with key sociodemographic trends among PWH in the United States. These parallels suggest that our findings may be applicable to subsets of the broader PWH population, particularly those disproportionately affected by intersecting socio-economic and racial disparities. Most of our participants identified as Black or African American, consistent with national data showing that nearly half of PWH belong to this group[57]. The predominance of participants reporting annual incomes below $20,000, which is nearly double the proportion of individuals living below the federal poverty level ($15,060 per year) compared to the national estimate of one-third of PWH, may reflect competing priorities that made it challenging to fully engage with the intervention and may help explain the nonsignificant results observed[57]."

20) Trial limitations, addressing sources of potential bias, imprecision, and, if relevant, multiplicity of analyses

20-i) Typical limitations in ehealth trials

Typical limitations in ehealth trials: Participants in ehealth trials are rarely blinded. Ehealth trials often look at a multiplicity of outcomes, increasing risk for a Type I error. Discuss biases due to non-use of the intervention/usability issues, biases through informed consent procedures, unexpected events.

	1	2	3	4	5	
subitem not at all important	○	○	○	○	◉	essential

Clear selection

Your response is too large. Try shortening some answers.

Does your paper address subitem 20-i? *

Copy and paste relevant sections from the manuscript (include quotes in quotation marks "like this" to indicate direct quotes from your manuscript), or elaborate on this item by providing additional information not in the ms, or briefly explain why the item is not applicable/relevant for your study

"Another significant limitation is the reliance on self-reported adherence data in the control group, which is prone to bias. Self-reported measures, particularly in the context of health behaviors, can be subject to overreporting due to recall bias or social desirability, where participants may provide responses they believe are more acceptable to researchers[69, 70]. Although the SRSI scale demonstrated high internal consistency, discrepancies between self-reported adherence and CleverCap data suggest that participants' actual behaviors differed. Some adjusted their adherence schedules based on personal beliefs, indicating that self-reported data may not fully reflect true adherence.
Discrepancies between the planned and delivered intervention fidelity were observed, with several factors impeding full implementation. While the intervention was designed to provide consistent support through regular CHW sessions and timely CleverCap notifications, technical challenges such as unreliable local telecommunication networks affected the delivery of mHealth components, leading to missed or delayed notifications. Additionally, participant engagement waned over time, with some individuals disengaging from CHW sessions and underutilizing the CleverCap device, a challenge commonly reported in mHealth interventions[71, 72]. The older demographic of the study population introduced additional barriers, as many participants had difficulty programming medication alerts without CHW assistance, leading to missed doses. This aligns with prior research indicating that older adults frequently encounter usability issues with electronic adherence tools, which can negatively impact adherence[71]. These findings highlight the importance of tailoring adherence interventions to address technological literacy and providing structured training for older adults."

21) Generalisability (external validity, applicability) of the trial findings

NPT: External validity of the trial findings according to the intervention, comparators, patients, and care providers or centers involved in the trial

Your response is too large. Try shortening some answers.

21-i) Generalizability to other populations

Generalizability to other populations: In particular, discuss generalizability to a general Internet population, outside of a RCT setting, and general patient population, including applicability of the study results for other organizations

	1	2	3	4	5	
subitem not at all important	○	○	○	○	◉	essential

Clear selection

Does your paper address subitem 21-i?

Copy and paste relevant sections from the manuscript (include quotes in quotation marks "like this" to indicate direct quotes from your manuscript), or elaborate on this item by providing additional information not in the ms, or briefly explain why the item is not applicable/relevant for your study

"This study has several limitations that should be considered. The small sample size of 40 participants limited the statistical power, making it difficult to detect significant differences between groups and reducing the generalizability of findings to broader populations of PWH. Furthermore, the small sample size for positive outcome events, such as viral suppression at follow-up, may have affected the reliability of the effect size estimate, particularly within the intervention group. The lower retention rate in the intervention group compared to the control group, while not statistically significant, suggests challenges in effectively engaging participants assigned to the intervention. This highlights the need to address structural and social determinants of health when designing ART adherence interventions. Future research should explore strategies to reduce these barriers, such as more flexible engagement options or additional resources.
Additionally, recruitment challenges particularly among younger PWH (18–29 years), were notable. Social media platforms used for advertising, such as POZ.com, primarily attracted older participants due to their demographic reach. Alternative platforms like TikTok, which are more commonly used by younger populations, should be prioritized in future campaigns[66]. Strategies such as peer-driven recruitment and culturally tailored messaging could help engage younger individuals who may be less trusting of traditional healthcare outreach methods. Addressing time constraints and providing more flexible scheduling options may also increase participation among this demographic[67, 68]. It is also important to note that participants who responded to advertisements on POZ.com may not be representative of the broader U.S. population living with HIV, introducing potential selection bias. Financial incentives for study participation may have influenced responses, potentially skewing the participant pool toward individuals with greater financial need."

Your response is too large. Try shortening some answers.

21-ii) Discuss if there were elements in the RCT that would be different in a routine application setting

Discuss if there were elements in the RCT that would be different in a routine application setting (e.g., prompts/reminders, more human involvement, training sessions or other co-interventions) and what impact the omission of these elements could have on use, adoption, or outcomes if the intervention is applied outside of a RCT setting.

	1	2	3	4	5	
subitem not at all important	○	○	○	◉	○	essential

Clear selection

Your response is too large. Try shortening some answers.

Does your paper address subitem 21-ii?

Copy and paste relevant sections from the manuscript (include quotes in quotation marks "like this" to indicate direct quotes from your manuscript), or elaborate on this item by providing additional information not in the ms, or briefly explain why the item is not applicable/relevant for your study

"Discrepancies between the planned and delivered intervention fidelity were observed, with several factors impeding full implementation. While the intervention was designed to provide consistent support through regular CHW sessions and timely CleverCap notifications, technical challenges such as unreliable local telecommunication networks affected the delivery of mHealth components, leading to missed or delayed notifications. Additionally, participant engagement waned over time, with some individuals disengaging from CHW sessions and underutilizing the CleverCap device, a challenge commonly reported in mHealth interventions[71, 72]. The older demographic of the study population introduced additional barriers, as many participants had difficulty programming medication alerts without CHW assistance, leading to missed doses. This aligns with prior research indicating that older adults frequently encounter usability issues with electronic adherence tools, which can negatively impact adherence[71]. These findings highlight the importance of tailoring adherence interventions to address technological literacy and providing structured training for older adults.
Lastly, the lack of a universal data verification process for the DBS results posed a limitation. While DBS samples were collected and processed according to established protocols, most participants' DBS results were not cross verified with other sources. Only a small subset of participants, who had requested a screening visit and subsequently provided electronic health record viral load data, had their DBS results cross-checked. This reliance on unverified DBS data for most participants may introduce potential inaccuracies. Furthermore, the moderate sensitivity (80–95%) and specificity (85–90%) of DBS for viral load thresholds, such as 200 - 1,000 copies/mL, could lead to misclassification, with some participants incorrectly identified as having high or low viral loads[73-75]. Future studies should incorporate systematic verification processes, such as routine cross-checking with laboratory results or electronic health records, to enhance the reliability of biomarker data."

OTHER INFORMATION

23) Registration number and name of trial registry

Your response is too large. Try shortening some answers.

Does your paper address CONSORT subitem 23? *

Copy and paste relevant sections from the manuscript (include quotes in quotation marks "like this" to indicate direct quotes from your manuscript), or elaborate on this item by providing additional information not in the ms, or briefly explain why the item is not applicable/relevant for your study

ClinicalTrials.gov ID: NCT05938413

24) Where the full trial protocol can be accessed, if available

Does your paper address CONSORT subitem 24? *

Cite a Multimedia Appendix, other reference, or copy and paste relevant sections from the manuscript (include quotes in quotation marks "like this" to indicate direct quotes from your manuscript), or elaborate on this item by providing additional information not in the ms, or briefly explain why the item is not applicable/relevant for your study

Wood, O.R., et al., A community health worker and mobile health app intervention to improve adherence to HIV medication among persons with HIV: the CHAMPS study protocol. BMC Public Health, 2023. 23(1): p. 942.

https://reporter.nih.gov/search/9JnPJH6eJkOfBbczG9Dfaw/project-details/9235576

25) Sources of funding and other support (such as supply of drugs), role of funders

Your response is too large. Try shortening some answers.

Does your paper address CONSORT subitem 25? *

Copy and paste relevant sections from the manuscript (include quotes in quotation marks "like this" to indicate direct quotes from your manuscript), or elaborate on this item by providing additional information not in the ms, or briefly explain why the item is not applicable/relevant for your study

"This study was supported by the National Institute of Nursing Research (NINR) under award number R01NR019758. DBS kit processing at the Miriam Hospital (Providence, RI) was supported by the Providence-Boston Center for AIDS Research under award number P30AI042853. "

X27) Conflicts of Interest (not a CONSORT item)

X27-i) State the relation of the study team towards the system being evaluated

In addition to the usual declaration of interests (financial or otherwise), also state the relation of the study team towards the system being evaluated, i.e., state if the authors/evaluators are distinct from or identical with the developers/sponsors of the intervention.

	1	2	3	4	5	
subitem not at all important	○	○	○	○	◉	essential

Clear selection

Does your paper address subitem X27-i?

Copy and paste relevant sections from the manuscript (include quotes in quotation marks "like this" to indicate direct quotes from your manuscript), or elaborate on this item by providing additional information not in the ms, or briefly explain why the item is not applicable/relevant for your study

"The authors declare that they have no conflicts of interest to disclose."

Your response is too large. Try shortening some answers.

About the CONSORT EHEALTH checklist

As a result of using this checklist, did you make changes in your manuscript? *

- ○ yes, major changes
- ◉ yes, minor changes
- ○ no

What were the most important changes you made as a result of using this checklist?

Your answer

How much time did you spend on going through the checklist INCLUDING making changes in your manuscript *

Time spent on the checlist and making manuscript changes: ~1.5 hours

As a result of using this checklist, do you think your manuscript has improved? *

- ◉ yes
- ○ no
- ○ Other:

Your response is too large. Try shortening some answers.

Would you like to become involved in the CONSORT EHEALTH group?

This would involve for example becoming involved in participating in a workshop and writing an "Explanation and Elaboration" document

- yes
- no
- Other:

Any other comments or questions on CONSORT EHEALTH

Your answer

STOP - Save this form as PDF before you click submit

To generate a record that you filled in this form, we recommend to generate a PDF of this page (on a Mac, simply select "print" and then select "print as PDF") before you submit it.

When you submit your (revised) paper to JMIR, please upload the PDF as supplementary file.

Don't worry if some text in the textboxes is cut off, as we still have the complete information in our database. Thank you!

Final step: Click submit !

Click submit so we have your answers in our database!

Submit Clear form

Never submit passwords through Google Forms.

This content is neither created nor endorsed by Google. - Terms of Service - Privacy Policy

Does this form look suspicious? Report

Your response is too large. Try shortening some answers.

Your response is too large. Try shortening some answers.
